# Phenoxyethyl Piperidine/Morpholine Derivatives as PAS and CAS Inhibitors of Cholinesterases: Insights for Future Drug Design

**DOI:** 10.1038/s41598-019-56463-2

**Published:** 2019-12-27

**Authors:** Yaghoub Pourshojaei, Ardavan Abiri, Khalil Eskandari, Zahra Haghighijoo, Najmeh Edraki, Ali Asadipour

**Affiliations:** 1 Department of Medicinal Chemistry, Faculty of Pharmacy and Pharmaceutics Research Center, Kerman University of Medical Sciences, Kerman, Iran; 2 Neuroscience Research Center, Institute of Neuropharmacology, Kerman University of Medical Sciences, Kerman, Iran; 3 Medicinal and Natural Products Chemistry Research Center, Shiraz University of Medical Sciences, Shiraz, Iran

**Keywords:** Chemical biology, Small molecules, Synthetic biology

## Abstract

Acetylcholinesterase (AChE) catalyzes the conversion of Aβ peptide to its aggregated form and the peripheral anionic site (PAS) of AChE is mainly involved in this phenomenon. Also catalytic active site (CAS) of donepezil stimulates the break-down of acetylcholine (ACh) and depletion of ACh in cholinergic synapses are well established in brains of patients with AD. In this study, a set of compounds bearing phenoxyethyl amines were synthesized and their inhibitory activity toward electric eel AChE (eeAChE) and equine butyrylcholinesterase (eqBuChE) were evaluated. Molecular dynamics (MD) was employed to record the binding interactions of best compounds against human cholinesterases (hAChE and hBuChE) as well as donepezil as reference drug. *In vitro* results revealed that compound **5c** is capable of inhibiting eeAChE activity at IC_50_ of 0.50 µM while no inhibitory activity was found for eqBuChE for up to 100 µM concentrations. Compound **5c**, also due to its facile synthesis, small structure and high selectivity for eeAChE would be very interesting candidate in forthcoming studies. The main interacting parts of compound **5c** and compound **7c** (most potent eeAChE and eqBuChE inhibitors respectively) with receptors which confer selectivity for AChE and BuChE inhibition were identified, discussed, and compared with donepezil’s interactions. Also during MD simulation it was discovered for the first time that binding of substrates like donepezil to dual CAS and PAS or solely CAS region might have a suppressive impact on 4-α-helical bundles near the tryptophan amphiphilic tetramerization (WAT) domain of AChE and residues which are far away from AChE active site. The results proposed that residues involved in donepezil interactions (Trp86 and Phe295) which are located in CAS and mid-gorge are the mediator of conformational changes in whole protein structure.

## Introduction

**Figure 1:**
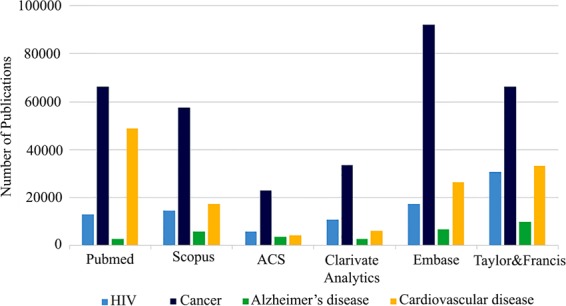
Results of some highly credible scientific engines with the keyword of “disease drug design” e.g. “Alzheimer drug design” or “cardiovascular drug design”.

According to the World Alzheimer Report 2018, every three seconds, one new case of dementia would be raised and about 50 million people were living with dementia in 2018 which researchers anticipate that this number would be triple by 2050^1^. Alzheimer’s disease (AD) accounts for more than two-thirds of all cases of dementia, the 5^th^ largest cause of mortality worldwide in 2018^2^. The dearth of AD literature compared with cardiovascular diseases, cancer and HIV along with the lack of the corroborated physiopathological mechanisms underlying this human disease have led to inadequate effective treatment strategies for AD (Fig. 1).

AChE accelerates the progression of AD by two possible mechanisms. First, the catalytic active (or anionic) site (CAS) of AChE is responsible for degradation of acetylcholine (ACh). According to the cholinergic hypothesis, an average 55% decrease in cholinergic signaling is observed in the brain of patients with AD compared with normal individuals. Moreover, cholinergic neurotransmission is involved in the memory consolidation and learning. These facts suggest that cholinergic modulation would be a beneficial strategy for treatment of AD^3^. Besides, the peripheral anionic site (PAS) generates a stable complex with *β*-amyloid peptide (A*β*) and by this means expedite the oligomerization of A*β* peptides and aggregation of senile plaques^4,5^. Incubation of AChE with A*β* peptide results in three times greater aggregation than A*β* alone^6^. Impressively, edrophonium, a CAS inhibitor, fails to slow down the A*β* aggregation whereas propidium, a PAS inhibitor (which is experimentally confirmed by X-ray crystallography that is selective for PAS and doesn’t interfere with CAS^7^) reduces the aggregation^8^. Propidium has an IC_50_ of 34.6 µM compared with 5.36 µM for edrophonium^9^. The emerging number of papers regarding the role of PAS in *β*-amyloid aggregation guided us toward dual CAS and PAS inhibitors and even selective PAS inhibitors instead of CAS inhibitors^10,11^.

It is suggested that PAS can facilitate the movement of ACh toward CAS, and therefore leads to boost the catalytic performance of AChE. This theory is further supported by the fact that AChE is one of the most efficacious biocatalysts in terms of kinetics^12^ and its kinetics of action is essentially limited by diffusion. Thus, dual inhibition of PAS and CAS of cholinesterases, not only restrain the rate-limiting step (diffusion of ACh to CAS), but also reduce the turnover number (K_cat_) of enzymes^13^. The CAS and PAS structural patterns are also certified in BuChE structure, as expected by 65% homology between AChE and BuChE sequences^14^.

The behavior of AChE and BuChE is more perplexing when previous surveys suggest that binding of some substrates to PAS at low pH might indeed activate the function of cholinesterases via a PAS-induced conformational change^15^. Nowadays, computational methods as accurate, accessible, trustworthy and reputable tools are extensively applied to study conformational changes of proteins structures. Molecular dynamics studies with sufficient simulation time have been recommended as reliable tools in assessment of these strange or obscure conformational changes^16–18^.

**Figure 2:**
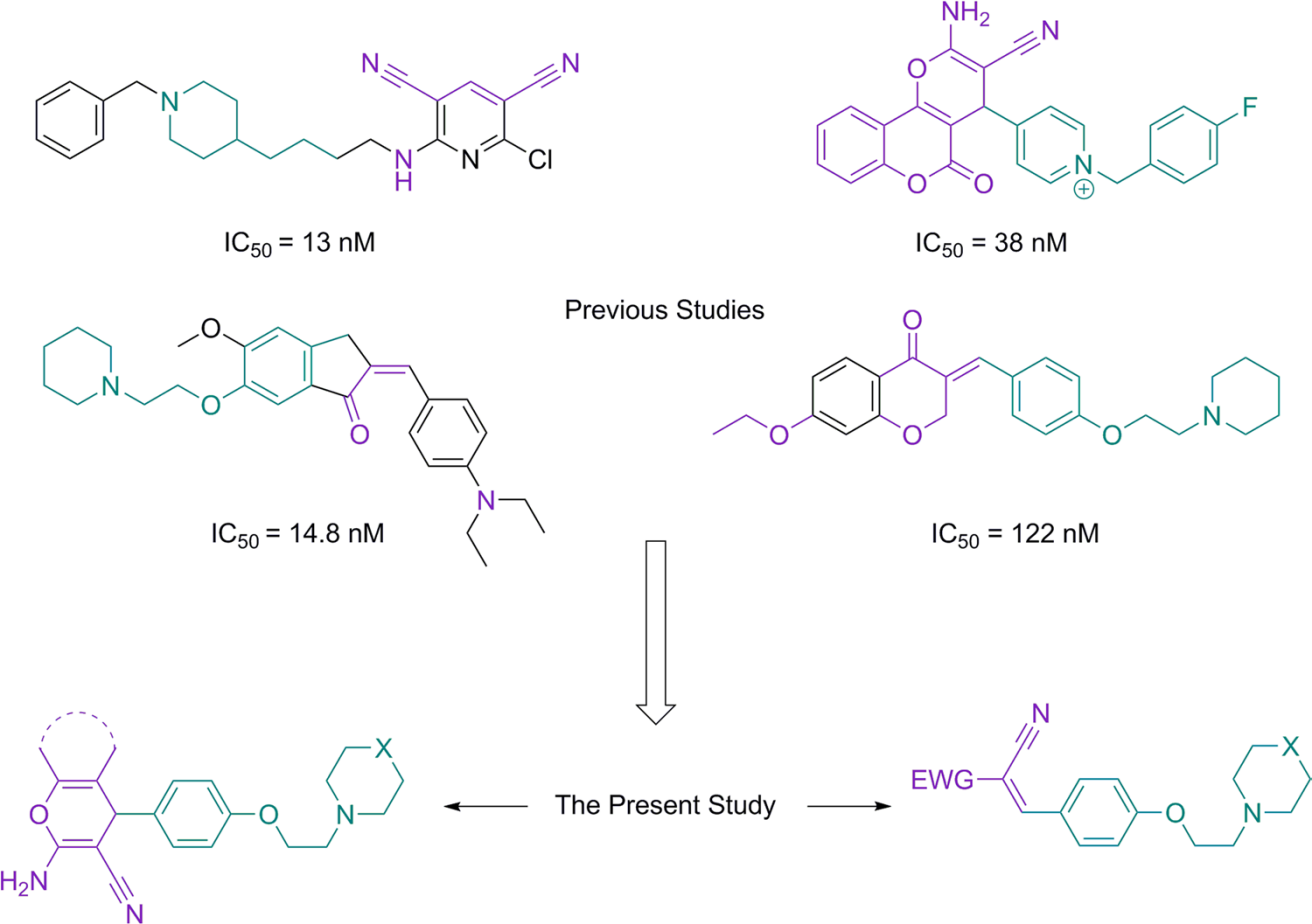
The rationale beyond the synthesis of new inhibitors of AChE^19,20,23^.

Previous studies have demonstrated that some core structures can bind and potently inhibit the AChE activity^19^. It is unraveled that an amine (usually in the form of piperidine) interacts with the anionic site of CAS and substituting the benzyl piperidine part of donepezil with phenoxyethyl piperidine or morpholine is one of the recent approaches toward stronger cholinesterase inhibitors^20,21^. In the other side of the molecule, nitrile groups, ketones and also etheric linkages are often observed^22–24^. These two parts are usually linked together by a phenyl or indanone scaffold^25^ (Fig. 2).

In this study, in connection with our previous studies on design, and synthesis of medicinally important compounds^26–28^, we designed, and synthesized new derivatives of piperidinylethoxy and morpholinoethoxy benzaldehyde by tandem Knoevenagel–Michael reactions to obtain new products based on structure similarities with previously characterized AChE and BuChE inhibitors as depicted in Fig. 2. In addition, anticholinesterase activity of these compounds was assessed by a modified Ellman’s method. We further analyzed our best results from *in vitro* evaluations by *in silico* molecular modeling studies. Finally we computationally assessed the pharmacokinetics profile of our best compounds and compared them with donepezil. Molecular dynamics (MD) used for *in silico* assessment of ligand-protein interactions in comparison with donepezil, illuminates the structure activity relationship (SAR) of our compounds for CAS and PAS and gives us new horizon for future drug design hereupon.

## Results and Discussion

### Chemistry

**Scheme 1:**
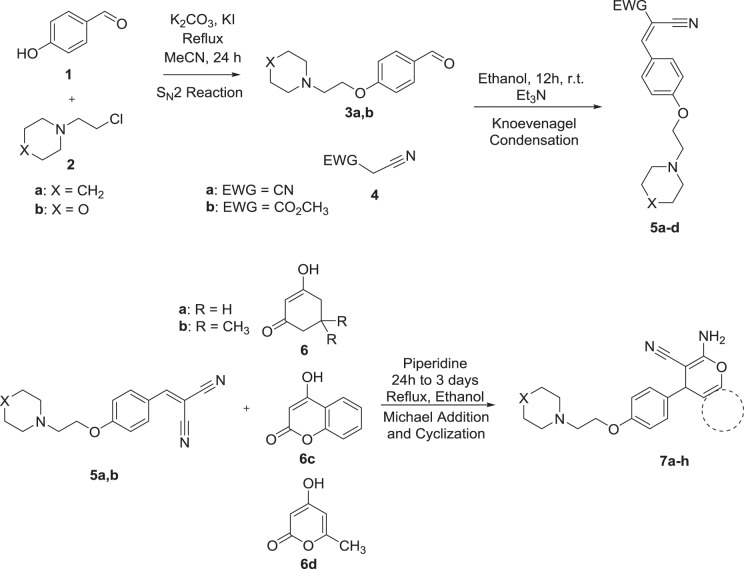
The synthetic steps to obtain target products.

The aim of the synthesis procedure of this study was to extend our knowledge about new hybridized scaffolds (by employing piperidinylethoxy, morpholinoethoxy and functionalized pyranocyclohexanone segments) which all are emerging as potent anticholinesterase agents. In this study, eight novel derivatives of 4*H*-pyran analogous (from Michael addition reactions and subsequent cyclization) and four known Knoevenagel products were synthesized. The first S_N_2 reaction for synthesis of aldehydes **3a,b** as precursor was started from reaction between 4-hydroxybenzaldehyde (**1**), and 1-(2-chloroethyl)piperidine (**2a**), 4-(2-chloroethyl)morpholine (**2b**) according to the literature^21^. Next, Knoevenagel products **5a–d** were synthesized with a highly efficient procedure (80–90%) from reaction between aryl aldehydes bearing *p*-alkoxyamine side chains **3a,b** with malononitrile (**4a**) or methyl cyanoacetate (**4b**). Finally, compounds **7a–h** as Michael addition products were achieved from reaction of **5a,b** with appropriate Michael donors **6a–d** in 50–70% yield (Scheme 1). It was observed that the yield of products **6a–d** were relatively proportional to nucleophilic potency of Michael donors.

### Evaluation of AChE and BuChE inhibitory activities

The inhibitory activity of synthesized compounds against eeAChE and eqBuChE was measured using the modified Ellman’s spectrophotometric assay. The obtained IC_50_ values of all designed derivatives in comparison with donepezil as reference drug are listed in Table 1. Analogs **3b**, **5b** and **7f** bearing morpholino pendant groups did not show any cholinesterase inhibitory activity against both enzymes. Eight compounds **5a**, **5c**, **5d** and **7a–e** exhibited good inhibitory activity against eeAChE with IC_50_ values in the range of 0.5–71.7 µM and also the range of IC_50_ values for six active compounds **3a**, **5d**, **7a–c** and **7 g** against eqBuChE are 2.5–71.3 µM. Structure-activity relationship (SAR) revealed that the presence of piperidinyl moiety seems to be play an important role in activity against eeAChE. Among the synthesized derivatives, compound **5c** with IC_50_ value of 0.5 ± 0.05 µM was the most potent derivative in this series. Comparing the effect of the piperidinyl versus morpholino pendant groups against eeAChE in compounds **5c** and **5d** clearly revealed that piperidinyl moiety can selectively inhibit eeAChE enzyme. In addition compounds **5a** (IC_50_ = 2.8 ± 0.05 µM) and **7e** (2.1 ± 0.05 µM) were found to have potent inhibitory activity whiles these compounds show no inhibitory activity for eqBuChE up to 100 µM concentrations. It is evident from the results of inhibitory activity against eqBuChE that the most potent compound was **7c** with IC_50_ values of 2.5 ± 0.6 µM which indicated eeAChE inhibition at IC_50_ values of 35.6 ± 3.7 µM. Thus this compound may be used as dual inhibitor for both enzymes. Similarly compound **7a** as Michael addition analogue containing piperidinyl counterpart was found to be another dual inhibitor of eeAChE (IC_50_ = 20.4 ± 9.3 µM) and eqBChE (IC_50_ = 8.1 ± 0.5 µM).

**Table 1:** IC_50_ of the series of products obtained in this study for inhibition of AChE and BuChE.

Compound	eeAChE IC_50_ µM	eqBuChE IC_50_ µM
	>100	71.3 ± 10.7
	>100	>100
	2.8 ± 1.2	>100
	>100	>100
	0.5 ± 0.05	>100
	15.8 ± 6.90	45.9 ± 9.3
	20.4 ± 9.3	8.1 ± 0.50
	59.2 ± 5.3	68.9 ± 2.3
	35.6 ± 3.7	2.5 ± 0.6
	71.7 ± 9.1	>100
	2.1 ± 1.2	>100
	>100	>100
	39.7 ± 11.2	61.7 ± 8.4
	45.9 ± 9.5	>100
	0.032	50

It is suggested that BuChE can have non-enzymatic functions related to neurodegenration observed in AD. Indeed, BuChE inhibits the fibrilization of monomeric A*β* to neurotoxic oligomers^29^. Also the greater rate of side-effects related to rivastigmine (a potent inhibitor of BuChE) versus donepezil (a mild inhibitor of BuChE) despite a relative improvement results in patients complicates the process of drug development for AD^30^. These findings suggest that although rivastigmine can raise the level of ACh in the brain of patients with AD, destroying other non-enzymatic function of this protein may cancel out the total effect and therefore the same final results are obtained for both donepezil and rivastigmine. Hence, an ideal anti-alzheimer agent should inhibit AChE (probabely both PAS and CAS) activity potently but should not impede BuChE activity. Fortunately, compound **5c** is more than 200 fold selective for AChE than for BuChE and based on our current understanding of AD physiopathology demonstrates a distinguished pharmacodynamics profile for AChE and BuChE.

As Knoevenagel condensation reaction is a very popular reaction in organic chemistry, compound **5c** indicates a novel and facile way for synthesizing new and highly selective AChE inhibitors. Furthermore, it is noteworthy to mention that the antioxidant activity of the synthesized compounds was also assessed by DPPH assay but unluckily no antioxidant activity was found for up to 100 µM concentration for neither of them.

### Kinetic study

**Figure 3:**
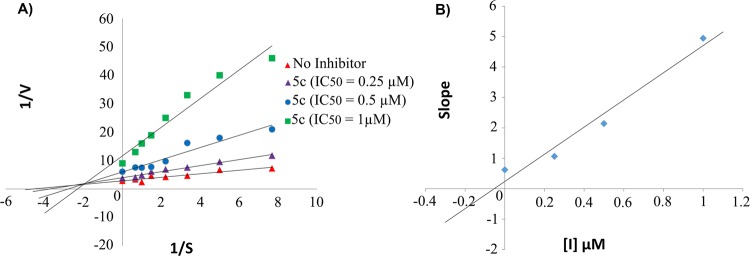
Kinetic study on the mechanism of eeAChE inhibition by compounds **5c**. (**A**) Overlaid Lineweavere-Burk reciprocal plot of eeAChE initial velocity at increasing ATCI concentration in the absence of inhibitors and in the presence of **5c** is shown. (**B**) The plot of the slopes against inhibitor concentration for calculation of Ki.

Enzyme kinetic analysis of the most potent derivative with highest inhibitory activity against, eeAChE (**5c**) was determined by Lineweaver-Burk plots (Fig. 3A). Two fixed concentrations of the inhibitor (IC_50_, 2IC_50_) were chosen and for each concentration, the initial velocity (V) of the substrate ATCI/BTCI hydrolysis was measured at different concentrations in the range of 0–1.5 mM. The results from the graphical analysis indicated that slopes and intercepts were increased with higher inhibitor concentrations. It can be presumed that the type of inhibition for the potent compound is a predominantly mixed-type inhibition which the results are compatible with what obtained from molecular dynamics studies. Furthermore, Ki value was obtained by extrapolating the slopes of Lineweaver-Burk plots against inhibitor concentrations (Fig. 3B). The result exhibited that Ki value for compound **5c** was 0.053 µM. This value of Ki outperforms many highly potent mixed-type inhibitors discovered recently^13,31,32^.

**Figure 4:**
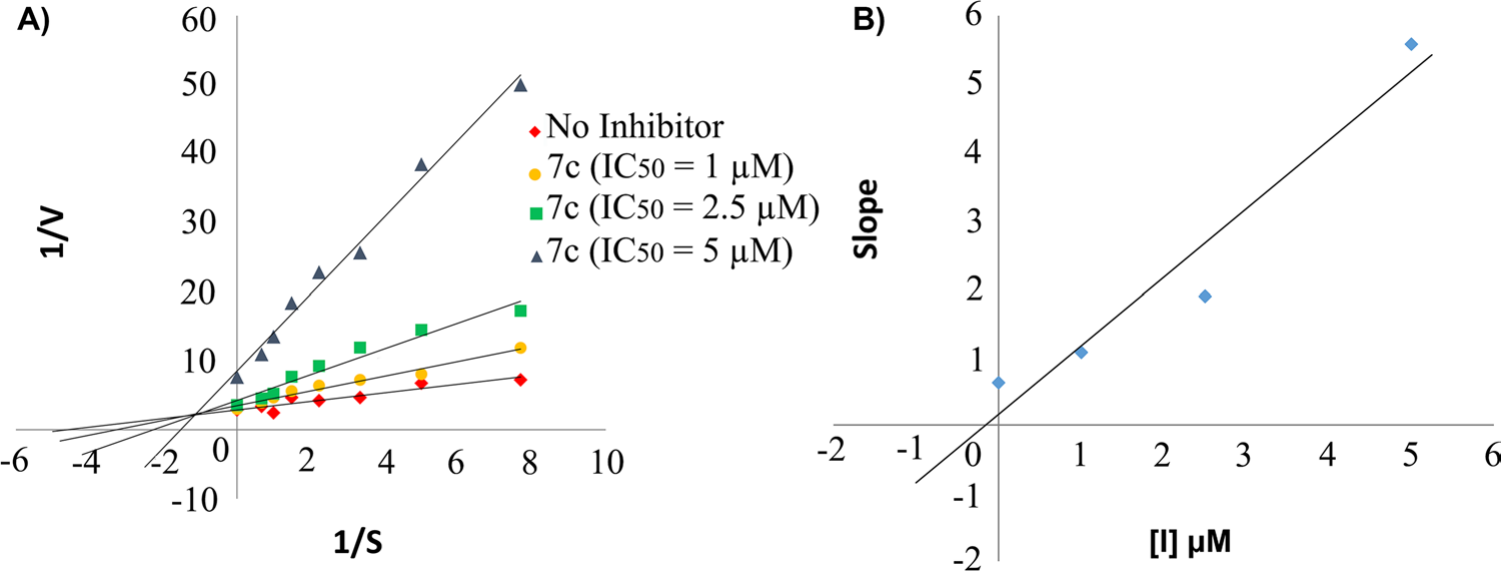
Kinetic study of eqBuChE inhibition by compound **7c**. (**A**) Overlaid Lineweavere-Burk reciprocal plot of eqBuChE initial velocity based on increasing BTCI concentration in the absence and in the presence of **5c** is depicted. (**B**) The plot of the slopes against inhibitor concentration for obtaining Ki value of compound **7c**.

Extrapolation of The plot Lineweavere-Burk reciprocal plot also yielded a mixed-type of inhibition for eqBuChE by compound **7c** (Fig. 4A). The plot of slopes against inhibitor concentration was also assessed for compound **7c**, the best eqBuChE inhibitor of this study and Ki value of 0.156 µM was observed (Fig. 4B).

### Molecular dynamics studies

**Figure 5:**
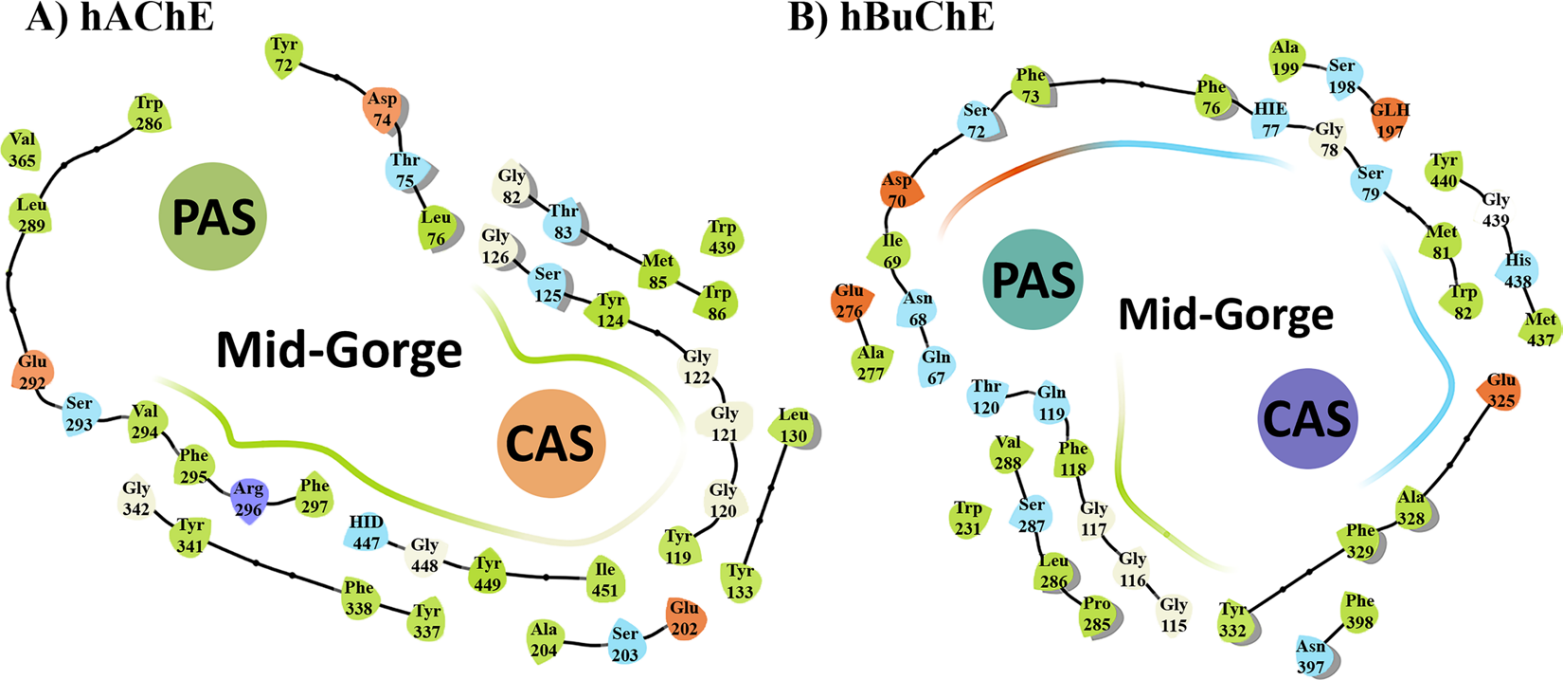
(**A**) CAS, PAS and mid-gorge pocket of human AChE (hAChE) (4EY7). The abundance of aromatic amino acids is significantly high especially in CAS. (**B**) Human BuChE (hBuChE) also share the same 3D-structure in the catalytic region, but contains more hydrophilic and charged residues than AChE (particularly in PAS and mid-gorge site).

**Figure 6:**
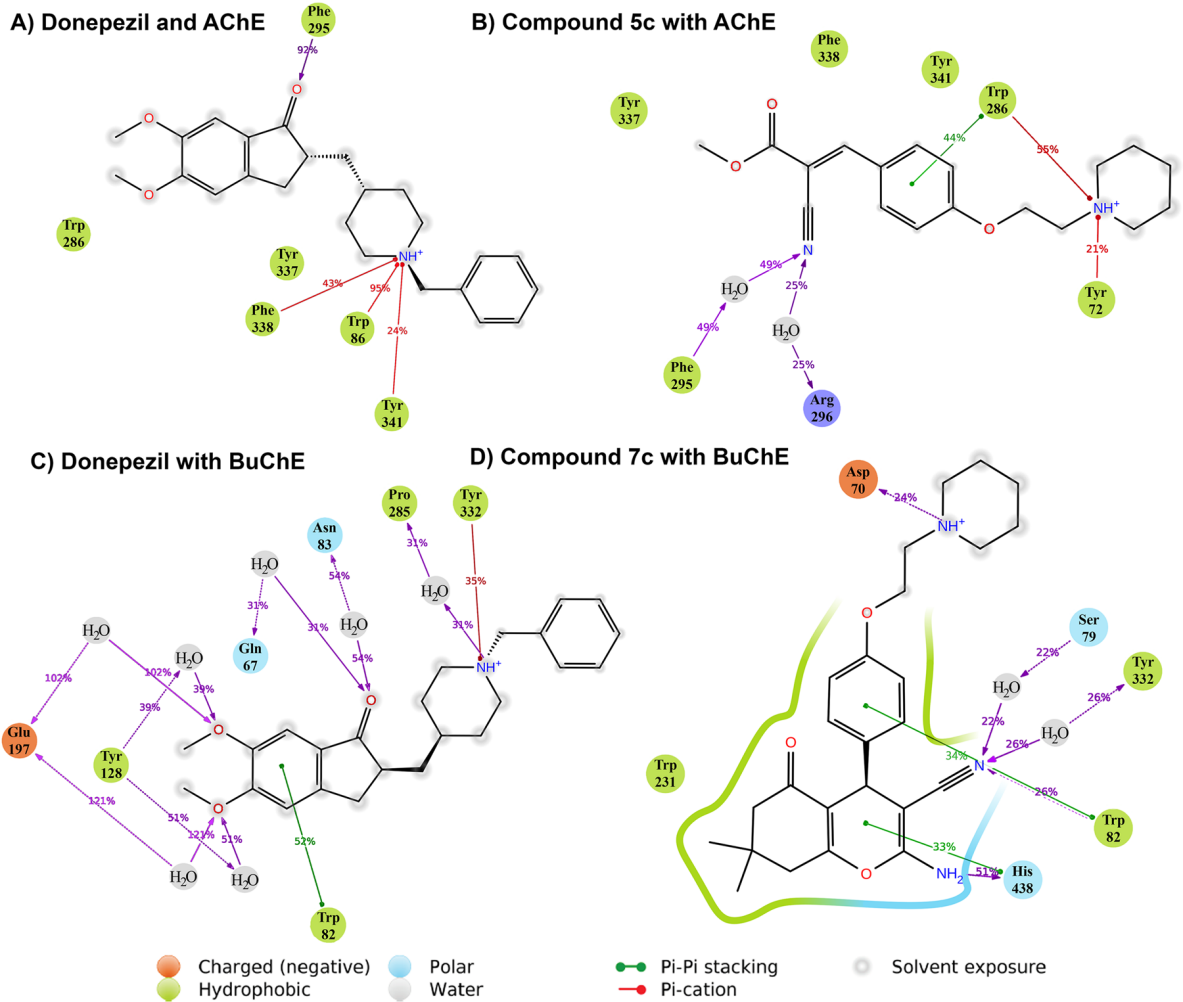
Binding interactions of donepezil and compound **5c** to hAChE (**A,B**); and donepezil and compound **7c** with hBuChE (**C,D**).

Considering the fact that there is high similarity in the binding pocket of eeAChE and hAChE, and as our compounds and their future derivatives are going to be evaluated as human therapeutics for treatment of Alzheimer’s disease, human enzymes (hAChE and hBuChE) were used for computational studies^33–36^. Molecular modeling studies disclosed the major binding interactions between our best compounds based on *in vitro* results with eeAChE and eqBuChE. For hAChE, it was not surprising that the key piperidine segment of both donepezil and compound **5c** exhibited the most important interactions. The protonated amine of piperidine participated 95% of the time of simulation in a π-cation interaction with Trp86, Phe338 and Tyr341 which are among the CAS and mid-gorge residues (Figs. 5 and 6). In the human AChE, CAS region is surrounded by Trp86, Tyr119, Tyr124, Tyr133, Glu202, Ser203, Trp439, His447, Tyr449 together with a series of glycine residues and PAS is in contact with Trp286, Tyr341, Asp74, Val365, Tyr72, Thr75 and Leu289 residues. The mid-gorge site of hAChE is characterized by the Asp74, Leu76, Phe297, Phe338, Phe295 and Arg296 and its opening is about 10.6 Å wide (average distance between Leu76 and Phe297) but the whole gorge pocket is about 20 Å deep.

The next important interaction of donepezil was a hydrogen-bond observed between the ketone group of indanone ring and Phe295 at the mid-gorge recognition site 92% of the time of simulation. As can be seen from Fig. 6A, donepezil displayed a considerable amount of solvent exposure (indicated in gray spots). Notwithstanding, the RMSD and RMSF of this drug indicate very low fluctuations and this is because of these two main interactions which both last more than 90% of the time.

In the other hand, compound **5c** piperidine group interacts mainly with the amino acids in the opening chamber of hAChE pocket. Trp286 and Tyr72 for 55% and 21% of the time are engaged respectively (Fig. 6B). The phenyl ring of compound **5c** also creates a π-π interaction with Trp286, one of the key amino acids in PAS. The nitrile group contributes to the stabilization of the structure by creation of two water bridges Arg296 and Phe295 which are located at the mid-gorge. By a closer look at compound **5c** interactions with hAChE, it can be comprehended that methyl carboxylate fragment has not participated in any major interactions with hAChE pocket residues. Therefore, manipulation of this fragment toward more lipophilic substituents may be fruitful to obtain more potent hAChE inhibitors.

The protonated nitrogen of the piperidine moiety of donepezil displays about one third persistency (35%) in the interaction with the conserved tyrosine residue in CAS region of hBuChE i.e. Tyr332 (homologous to Tyr341 in hAChE) (Fig. 6C). Also, seven water bridges were found in overall simulation which indicate a more hydrophilic nature for BuChE pocket (especially in PAS). Glu197, Tyr128 and Gln67 in PAS along with Trp82 and Asn83 in mid-gorge and Pro285 in CAS contribute to water-bridges with donepezil. All polar functional groups of donepezil were involved in these water bridges. Compound **7c** exhibited a noticeable 24% hydrogen-bond with Asp70 (in PAS). His438 also provided a significant (51%) hydrogen-bond with –NH_2_ and the nitrile group displayed two water-bridges with Tyr332 and Ser79 in CAS and mid-gorge. The structure of our best compound for BuChE (Compound **7c**) seems to not interact with CAS region. The ketone group of the cyclohexane ring, displayed no major interactions throughout MD and therefore can be safely removed or replaced by suitable substituents for achieving greater affinity (Fig. 6D).

Our results are in agreement with previous AChE inhibitors reported by Fang *et al*. in which they also tested some substituted phenoxyethyl piperidine derivatives and found mixed type behavior against the enzyme. Moreover, as we observed in this study these compounds and previously similar derivatives tend to interact with both CAS and PAS region of AChE^37^.

RMSD diagrams of the simulation were consistent with the binding interactions (Supplementary Fig. S1). Donepezil as a potent inhibitor of AChE, resulted in very low fluctuations (0.4 Å for the total simulation, Fig. S1A) and our most potent compound had about 1 Å oscillations during the MD. Nonetheless, this level of inhibition in spite of its small structure is an indicator of a very compelling lead-like structure for further structural manipulations (Fig. S1B). Donepezil also solidified the structure of hAChE backbone more than compound **5c** and this conforms with the favorable binding properties of donepezil with hAChE.

One of the interesting observations of this study is the reversed orientation of compounds **5a–d** in compassion to **7a–h** in the active site. In compounds **7a–h** the piperidine ring tends to be in CAS region while in compounds **5a–d** tends to bind to the PAS region.

For BuChE, donepezil is a weaker inhibitor, so a large fluctuation observed (Fig. S1C). Compound **7c** MD results, were satisfactory and verified the *in vitro* findings. With a very few steep oscillations, most of the time the RMSD range varied for less than 0.4 Å (Fig. S1D). The overall fluctuation for the backbone of the proteins were the same for hBuChE structures and suggested the equilibrated structure of this compound (less than 2 Å and without no major conformational change).

**Figure 7:**
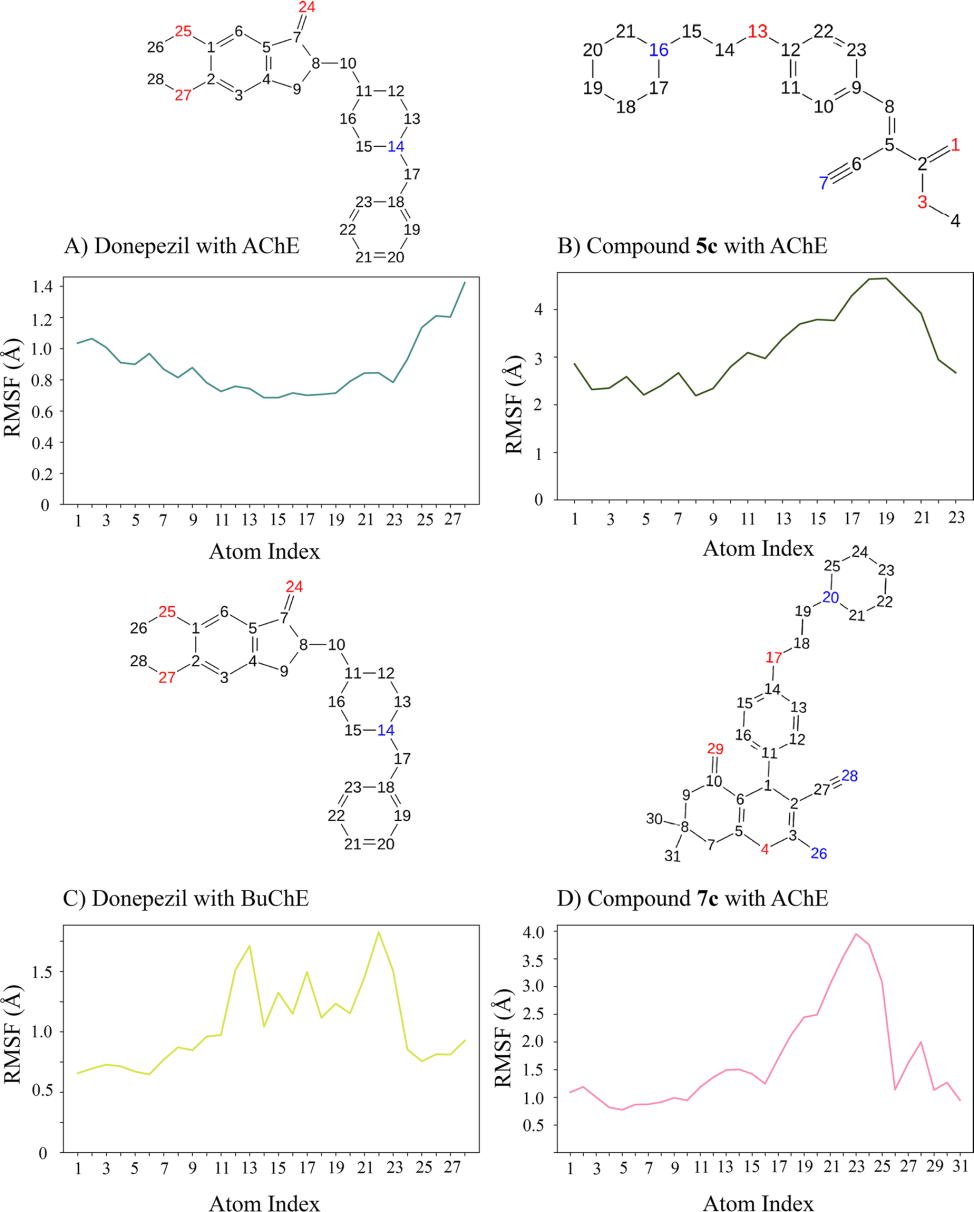
Ligand RMSF data for hAChE (**A,B**) and hBuChE (**C,D**), showing atoms with greatest and smallest fluctuations during molecular dynamics.

To analyze the average participation of each atom in the simulation, we surveyed the RMSF values of ligands. The benzyl piperidine part of donepezil illustrated the lowest RMSF value and the methoxy groups of the indanone ring displayed the highest. This does make sense because these methoxy groups did not supply any structural stabilization by intermolecular bonds (Fig. 7A). The key nitrogen (atom index 14), had the lowest value according to its 95% hydrogen-bond with Trp86. Compound **5c**, resulted in larger RMSF values especially for the far carbon atoms of the piperidine ring (atom index 19 and near carbon atoms, Fig. 7B). The *in vitro* results, suggested that substitution of this carbon atoms with oxygen (replacing piperidine moiety with morpholine) generates an inactive molecule against AChE. By comparing the experimental data with this finding, it is inferred that probably the nonspecific water-bridges and hydrogen-bonds displace this part of the molecule to the outer part of AChE. Therefore, it can be concluded that modification on the end part of these molecules is favorable for optimizing hit structures to a better lead molecule.

Our compounds demonstrated more satisfying RMSF properties regarding the hBuChE. In contrary to hAChE, the benzyl piperidine part of donepezil resulted in larger fluctuations than other atoms. One of the important results of this observation is that the benzyl piperidine part of donepezil confers this molecule selectivity toward AChE inhibition and likewise the methoxy groups in the indanone moiety provide BuChE selectivity (Fig. 7C). A comparison between the solvent exposure part of donepezil and RMSF results, proposes that alteration on the piperidine counterpart might lead more potent BuChE inhibitors. Similarly, for compound **7c**, the piperidine ring displayed the largest amount of RMSF. On the other hand, the –NH_2_ and nitrile groups yielded the lowest RMSF, consistent with interaction results (Fig. 7D).

**Figure 8:**
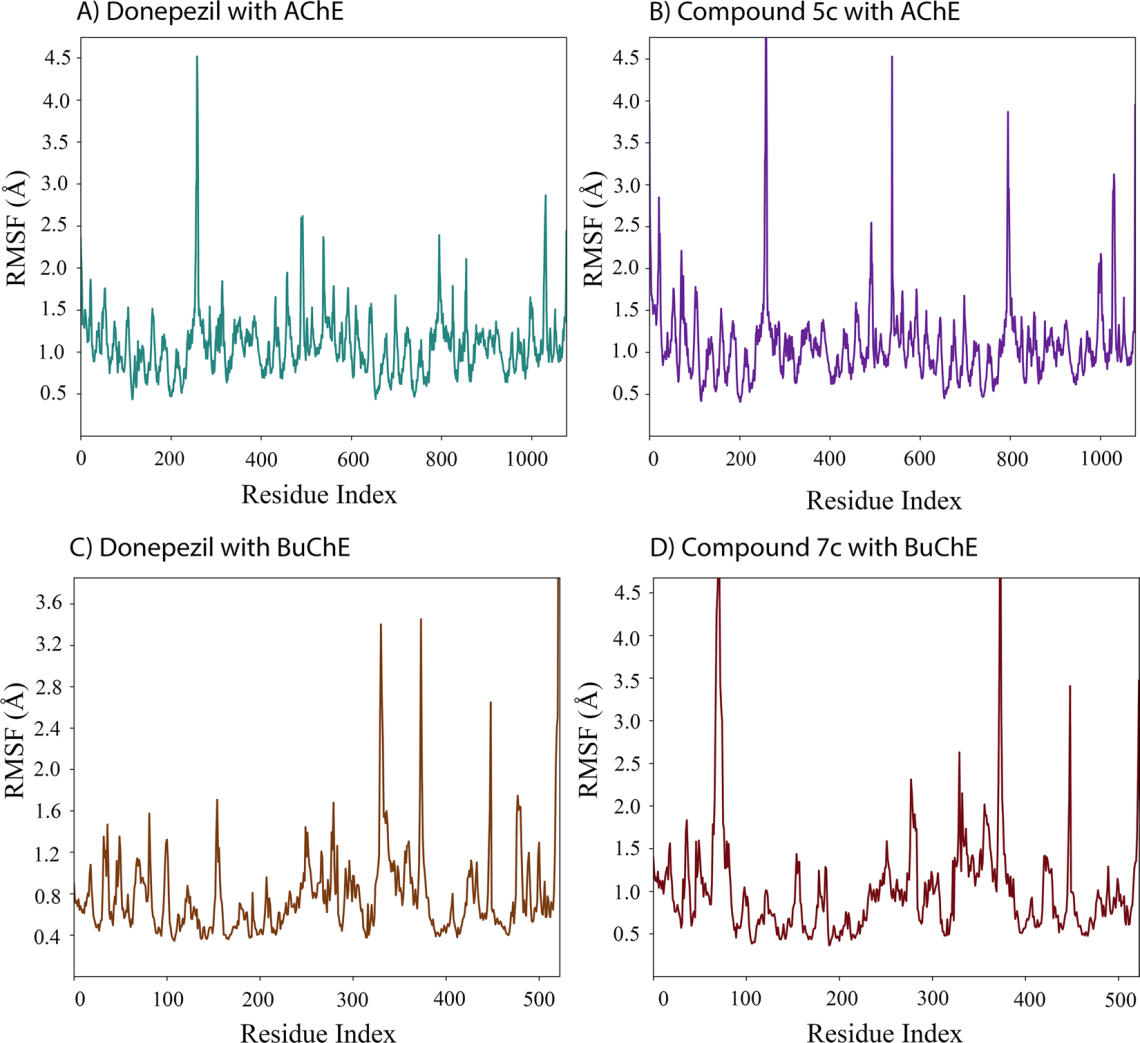
Protein RMSF, demonstrating the ligand’s ability to solidify and stabilize the structure of the protein during the computational simulation.

To assess the structural stability of the protein and its freedom of movement, RMSF diagrams of proteins were analyzed. Donepezil and compound **5c** resulted in nearly identical pattern except that two part of the protein (around residues 500 and 800) displayed larger jumps. AChE of this study has two subunits and therefore residues around 500, are related to the C-terminal of subunit A (Fig. 8A,B).

AChE as an example of morpheein protein structures, can be found in biological environment as monomer, dimer, tetramer as well as some other quaternary structures^38^. A comparison of protein fluctuations in the case of donepezil and compound **5c** suggests that residues involved in binding with donepezil suppress the conformational flexibility of other regions in the protein. Considering the large distance between the active site and these residues, this finding implies that binding of ligands to the catalytic site of AChE might suppress other conformational changes of the intact enzyme.

According to the previous studies, AChE is also an example of moonlighting proteins and plays key roles in synaptogenesis, formation of neural networks and cell adhesion^39^. It is hypothesized that laminin-1 (a scaffold glycoprotein involved in cell adhesion, migration and differentiation) interacts with AChE possibly indirectly through conformational modulation of PAS or via inhibiting of the entrance of ACh^40^. However, this study points out that the high fluctuation which is observed in the C-terminal of AChE, is suggestive of some other binding sites for proteins like laminin-1. The C-terminal of AChE (tryptophan amphiphilic tetramerization or WAT domain) is also thought to interact with the Proline-Rich Attachment Domain (PRAD) of Collagen-like Q subunit (ColQ). ColQ is another scaffold protein which associates AChE to synaptic basal lamina^41^. Donepezil suppressed the fluctuations near the C-terminal of each chain (before the WAT domain), which corresponds to four α-helical structures in total (for both chains) at the dimerization interface, indicates that binding of substrates to PAS or/and CAS might be a regulatory component in the non-classical functions of AChE (Fig. 8A). Since higher fluctuations were observed for compound **5c** in these helices and compound **5c** is more selective toward PAS region, it appears that CAS has the necessary amino acids for suppression of these fluctuations and not PAS independently. Compound **5c**, displayed a larger rate of fluctuations in other regions too, consistent with its larger IC_50_ values (Fig. 8B).

For hBuChE, the comparison between donepezil (Fig. 8C) and compound **7c** (Fig. 8D) show two different binding poses in the active site of the enzyme. Residues around 80 in BuChE display a lower fluctuation for donepezil. A mild decrease in fluctuations is observed near some residues around 300, where the donepezil interacting amino acids are present. Since compound **7c** is a more potent inhibitor of BuChE, the ability of compounds to suppress this region of the proteins might be a good indicator of BuChE inhibition.

AChE is distinctive from many other proteins in having three unique features; first, it is a moonlighting protein, possessing different non-classical functions in biological environment other than its classical catalytic activity. Second, it exists in morpheein forms, possibly by alternative splicing and interconverts between these monomeric, dimeric and tetrameric forms which might also be induced by conformational changes of binding substrates to its active site. This study confirmed that binding of substrates to PAS and CAS or only CAS region restrict the conformational flexibility of other parts, especially the four helical structures before the WAT domain. Third, it displays a peculiar deep gorge in the catalytic site, featuring two important binding sites i.e. CAS and PAS. This organization of amino acids makes AChE one of the most efficacious enzymes in terms of kinetics. Blocking the opening of this chamber, as achieved by PAS inhibitors, would be more gratifying due to dual blockade of catalytic activity and cholinesterase induced Aβ aggregation. Also it is well documented that synaptic AChE instigates the phosphorylation of tau (τ protein) which itself induces the activation of Glycogen Synthase Kinase 3β (GSK-3β) and overactivity of GSK-3β is linked to memory impairment and AD^5,42^. Though CAS has been characterized as the most probable relaying part of the enzyme, we should clearly indicate that Trp86 (CAS) and Phe295 (mid-gorge) are the most probable candidate residues which quench the fluctuations in other parts like C-terminal α-helices and probably parts involving in interaction with phosphorylation of tau. The proof for this theory lies on the fact that donepezil only inhibits the aggregation of Aβ peptide at high concentration and only by small amount (22% at 100 µM, as stated in introduction section) and MD results revealed that CAS and mid-gorge are the major sites of donepezil activity. Surprisingly, this apparently means that PAS accelerates the Aβ peptide aggregation whereas CAS (either directly or indirectly) induces the tau phosphorylation. This observation explains why CAS inhibitors like tacrine^43^ are able to mitigate the symptoms of AD in spite of their negligible inhibition of PAS region. Biochemical assays are needed to exactly assess the validity of this preliminary finding.

**Figure 9:**
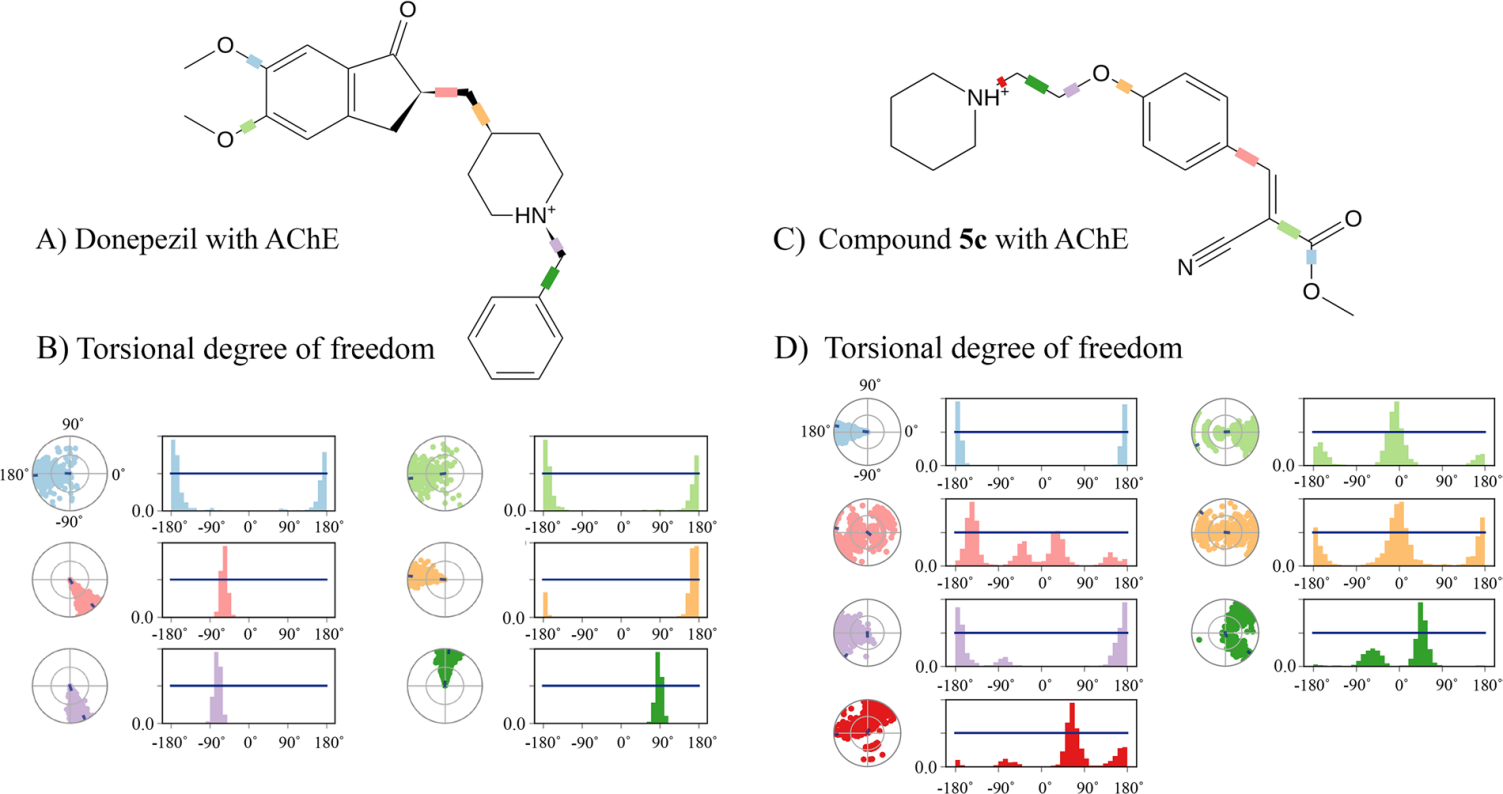
Contribution of each rotatable bond in the total molecular dynamics simulation. Bar plots represent the frequency of each torsions, and radial plots display the total number of occupied torsions during the simulation.

The ligand torsion profile obtained from MD studies assesses the conformational changes of each rotatable bond in the ligand structure, throughout simulation. The center of the radial plot is correlated with the beginning of the simulation and different conformations of each rotatable bond is recorded radially outwards by the evolution of simulation. The bar plots represent the probability density of each torsional rotation. A potent inhibitor of an enzyme occupies a rigid structure which can maintain the same binding orientation over the simulation and thus produces a narrower band and less variation. Analysis of ligand torsion profile gives insight for extracting the pharmacophore features necessary for interacting with critical residues in the proteins (Fig. 9).

The rotatable bonds between the phenyl and piperidine rings of donepezil showed the least changes during the simulation. This observation is in conformity with the role of protonated nitrogen in the CAS of hAChE as the central inhibitory fraction of the molecule. The other two rotatable bonds between the indanone ring and piperidine ring also indicated very low rotational variations during the simulation. This is also supported by the 92% binding interactions recorded for the ketone group of indanone and 95% of protonated amine. The two methoxy groups as suggested by RMSF analysis and binding interaction were among the most unfavorable moieties in the ligand structure and demonstrating different torsions due to lack of effective interactions with hAChE pocket residues (Fig. 9A,B).

The least variation in ligand torsion profile for compound **5c** rotatable bonds, were found in the carbon-nitrogen linkage of piperidine moiety. A closer look at the previous studies also confirms that this scaffold is also present in some other AChE inhibitors (Fig. 2)^19^. Variations in the other rotatable bonds of compound **5c**, especially in the electron withdrawing groups (EWGs) is suggestive of the lead-like and not drug-like properties of this compound. The compound **5c** is a Michael acceptor and therefore modification on this structure and cyclization might be a logical method for producing ligands with higher affinity (Fig. 9C,D).

**Figure 10:**
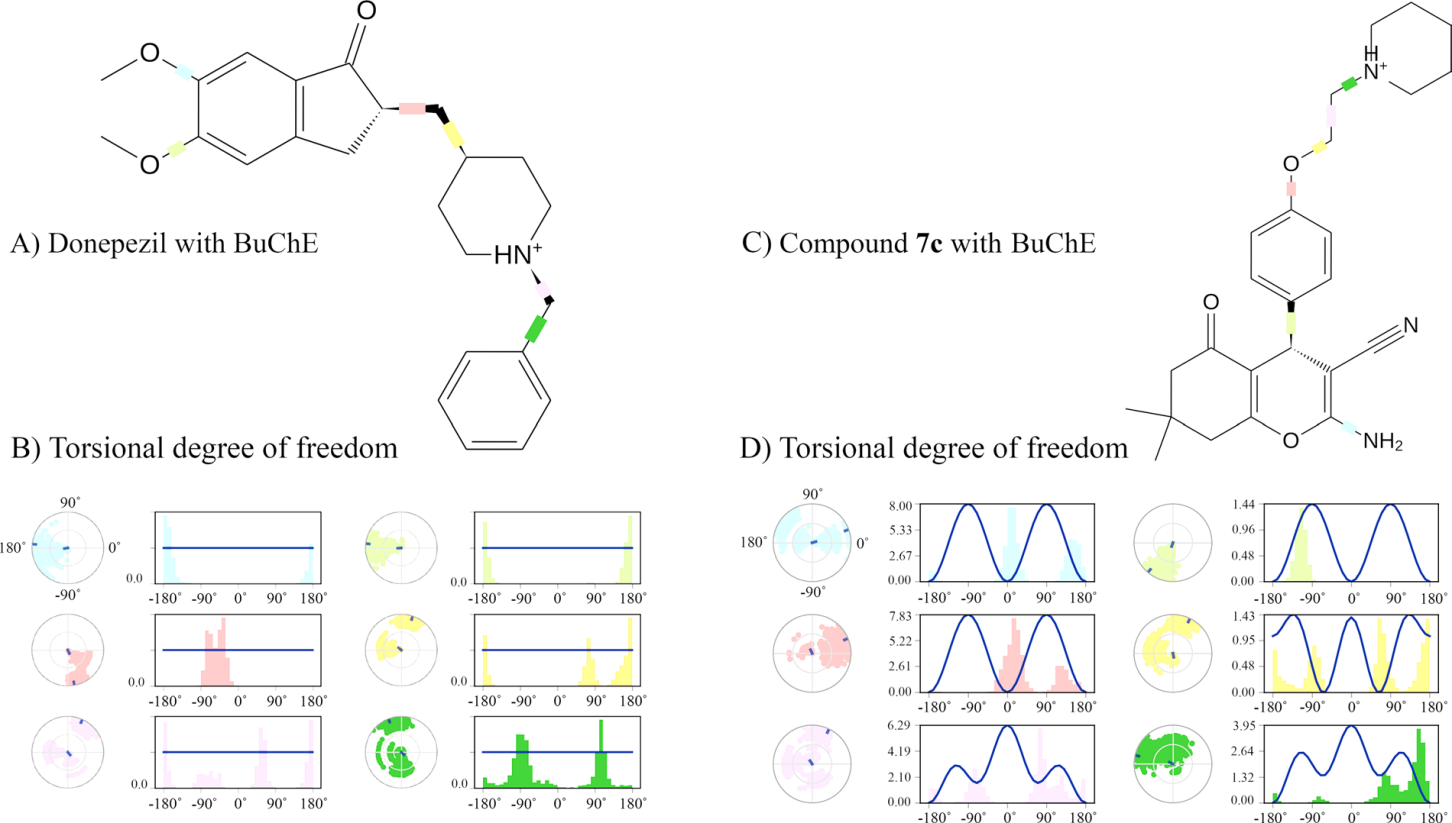
Torsional profile of donepezil and compound **7c** with hBuChE.

The ligand torsion profile for donepezil supports the idea that methoxy groups of the indanone ring are more necessary for the hBuChE inhibitors. The carbonyl group of indanone ring is also an important functional group for inhibiting hBuChE activity and as a result, the rotatable bonds which connect the benzyl piperidine moiety to indanone, display smaller variations (Fig. 10A,B). Compound **7c** was supposed to have the least torsional deviations for the rotatable bond which links the phenyl to the pyran ring, because of the persistent interaction of –NH_2_ and nitrile group. The same deduction is interpreted for –NH_2_ torsion with carbon group of the pyran ring (Fig. 10C,D).

Eventually, it is interesting to summarize the major pharmacophore features based on SAR stdudies obtained by MD and *in vitro* results:

1. Phenoxyethyl piperidine compounds bearing vinyl nitrile group in *para* position represent high selectivity for AChE inhibition and they mainly interact with PAS region of AChE.
2. 4-Phenyl-4*H*-pyran scaffold bearing primary amine and nitrile groups drives the selectivity of the molecule toward BuChE inhibition.
3. Though in this study we focused on the cyclic amines like morpholine and piperidine, based on MD results and previous studies, it seems that linear tertiary amines (rivastigmine-like structures) would be more potent candidates for future designs. This fact is strongly supported by two observations; first, replacing piperidine with morpholine in almost all cases results in lower inhibition and second, highest RMSF values are obtained from carbons which are on 4^th^ position of piperidine ring. Applying smaller rings like pyrolidine instead of piperidine might be another interesting idea.
4. Considering the large solvent exposure and the large middle width of AChE active site (10 Å), larger hydrophobic substituents in other regiens would be interesting for better interaction with PAS and CAS.

#### Prediction of pharmacokinetic parameters

*In silico* pharmacokinetic properties predicted by admetSAR and SwissADME servers^44,45^ for the synthesized compounds with respect to donepezil are presented in Table 2. The results revealed that compound **5c** displays high GI absorption, low topological polar surface area (TPSA) which is the sum of all polar surface areas of the molecule and necessary for penetration into brain^46^ and no carcinogenicity. The Knoevenagel products (as compound **5c**) display low metabolizing tendency by liver cytochromes but the products of Miachael addition demonstrated more susceptibility toward metabolization. All compounds passed the druglikeness rules (Lipinski’s rule of five, Ghose, Veber, Egan and Muegge rules). The pan assay interference compounds (PAINS), assess the promiscuity feature of hit compounds, capable of inducing positive results in many biochemical and pharmacological assays^47^. Our results indicate that compounds **5a–d** might be promiscuous in these assays, but compounds **7a–h** are negative for this feature. Compound **5c** is a Michael acceptor and due to its possible reactivity with biological molecules, it is included in the Brenk list of fragments with chemically unstable or toxic properties^48^.

**Table 2:** Pharmacokinetics profile of the synthesized cholinesterase inhibitors versus donepezil.

Compound	PAINS and Brenk^a^	BBB Permeant	Druglikeness (Violations)^b^	TPSA	GI Absorption^c^	Metabolism by CYPs^d^
Donepezil	0	Yes	0	38.77 Å²	High (0.55)	3A4, 2D6
**5c**	2	Yes	0	62.56 Å²	High (0.55)	2C9
**7c**	0	No	0	88.58 Å²	High (0.56)	2C9, 2C19, 2D6, 3A4

^a^Promiscuity of compounds. ^b^Violations of the structures from Lipinski’s rule of five, Ghose, Veber, Egan and Muegge. ^c^Gastrointestinal absorption and bioavailability score. ^d^Liver Cytochromes P450.

To summarize our *in silico* pharmacokinetics findings, it is concluded that compounds **5a–d** with high inhibitory activity against AChE can be regarded as a lead-like core for further research and compounds **7a–h** are drug-like structures, yet need to be pharmacokinetically and pharmacodynamically optimized. As also mentioned, removing the ketone group of cyclohexane ring and replacing the pyran ring with cyclohexane or phenyl ring might be interesting for future drug design. Generally, according to the structural nature of AChE and BuChE binding sites, Michael donors having more lipophilic nature might be more suitable for AChE inhibition and those with higher hydrophilicity are more appropriate for BuChE. SwissADME also predicts that because of having a molecular weight of more than 350 and XLogP >3.5 for donepezil and compound **7c**, they are not regarded as suitable lead-like structures.

In summary, our results indicate that the benzylpiperidine moiety of donepezil plus the ketonic indanone ring are responsible for selective inhibition of AChE and the methoxy groups attached to the indanone ring is responsible for the inhibition of BuChE selectively, and compounds bearing this amine scaffold are not conducive to design BuChE selective inhibitors. These important results can be used in future studies to provide more selective drugs with lower side-effects.

## Conclusion

In this study, a set of compounds bearing phenoxyethyl piperidine/morpholine side-chains were designed, synthesized and their biological effects against eeAChE and eqBuChE were evaluated. The results of our *in vitro* studies were significant considering that we identified a small molecule lead-like structure (compound **5c**) for eeAChE, which was highly selective for eeAChE (no inhibition for eqBuChE up to 100 µM concentrations were observed) and was capable of undergoing further chemical reactions with other nucleophiles to provide a more diverse set of chemicals. Even though compound **5c** indicated an IC_50_ comparable with some mixed-type inhibitors discovered previously, its 53 nM Ki value, introduced this compound as one of the most potent mixed-type inhibitors discovered for AChE (in terms of dissociation constant or Ki).

To explore the binding interactions and stability of ligands in the binding pocket and investigating the functional groups involved in ligand-protein complex, molecular dynamics was performed. The results of this study is of great interest because we compared the binding interactions of our compounds with cholinesterases obtained by molecular dynamics with the reference drug, donepezil. This comparison refined our understanding toward selective AChE and BuChE inhibitors. Our compounds tend to inhibit the PAS site of AChE more effectively than CAS. We also suggested that either dual PAS and CAS inhibition or CAS inhibition might have an impact on the C-terminal α-helical bundles in the vicinity of the WAT domain of AChE, which is mainly engaged in tetramerization of AChE. CAS and not PAS might also be involved in interaction of AChE with other proteins like ColQ and laminin-1. Although further studies are encouraged to more precisely test this theory, our results suggest that CAS might indirectly be involved in the phosphorylation of tau and alteration of AChE morpheein structures. We cannot be sure whether which interaction is affected mostly by binding of substrates to CAS, but it is evidently clear that CAS modulate the dynamics of other residues of AChE. Finally, the role of CAS in dynamic structure of AChE rehabilitates the use of CAS inhibitors for management of AD. AChE and BuChE inhibitors are among the candidate molecular targets in several other diseases like Parkinson’s disease, myasthenia gravis, glucoma and even autism^49^. Future application of cholinesterase inhibitors in pharmacological assays toward these diseases might disclose other potential capabilities of these molecules.

## Materials and Methods

### Chemistry

An electrothermal IA9100 apparatus was applied for measuring melting points of newly synthesized compounds and they are corrected. An FT-IR Tensor 27 infrared spectrophotometer manufactured by Bruker is used to record FT-IR spectra and also KBr salt is used as matrix. An FT-NMR Bruker Avance Ultra Shield Spectrometer (300 MHz for ^1^H and 75 MHz for ^13^C) was used to record NMR spectra while DMSO was applied as solvent. δ refers to the chemical shifts which are stated in part per million (ppm). Tetramethylsilane (TMS) was used as internal standard and all other signals are measured to its chemical shift (δ = 0 ppm, used for calibrating chemical shifts). J denotes coupling constants and is stated in Hz. Also, definition of used abbreviations for spin multiplicities are specified as follow: (s, d, t, m, q, br, and brs are referred to singlet, doublet, triplet, multiplet, quartet, broad, and broad singlet respectively. An Agilent 6224 TOF LC/MS was applied for high resolution mass spectra (HRMS). All chemicals, starting materials and solvents applied in the synthetic procedures were bought from Merck, Sigma-Aldrich or Across Organics commercial businesses. The progress of the reactions was checked by thin-layer chromatography (TLC plates equipped with precoated silica gel F_254_).

### General procedure for the synthesis of 4-(2-(piperidin-1-yl)ethoxy)benzaldehyde and 4-(2-morpholinoethoxy)benzaldehyde (compounds **3a**,**b**)

Compounds **3a,b** were prepared by slightly modification in what reported in the literature^21^. To this end, 4-hydroxybenzaldehyde (40 mmol, 5.49 g), was added to a single neck round bottom flask (capacity 500 mL) equipped with an oil bath containing potassium carbonate (122 mmol, 16.8 g, as the catalyst) and acetonitrile (160 mL, as solvent). It was allowed to the mixture to be refluxed for 2 h and then it cooled down to room temperature. Next, a catalytic amount of potassium iodide was added to the mixture. Afterward, aminoethyl chloride hydrochloride (40 mmol) was added and the mixture was refluxed again for about 24–48 h. The progress of the reaction was checked by TLC (chloroform/methanol as eluent; v:v/15:1). After the reaction was established and its completion was confirmed by TLC, the reaction mixture was filtered under suction to separate heterogeneous inorganic catalyst and the catalyst was washed with hot acetonitrile (3 × 60 mL). The filtrate was evaporated under reduced pressure to obtain solid residue. The pure product was obtained from silica column chromatography (methanol/dichloromethane as eluent; v/v: 1–9%) as ivory to yellowish solid in 50–60% yield.

### General procedure for the synthesis of compounds **5a–d**

The routine procedure to the synthesis of Knoevenagel products was addition of malononitrile (1.5 mmol, 0.099 g) and catalytic amount of triethylamine (Et_3_N) (10 mol%) to the purified aldehydes (1 mmol) in ethanol (for reactions with malononitrile) or methanol (for reactions with methyl cyanoacetate). After stirring the solution at room temperature (4 h), the reaction displayed no further progression and the sediments were filtered and recrystallized in ethanol or isopropyl alcohol to refine the final products. Progression of reaction was controlled by TLC, using EtOAc/n-hexane (v:v/1:1) as eluent. The Knoevenagel products were obtained in high efficient yields (80–90%)

### General procedure for the synthesis of compounds **7a–h**

The purified product of the previous Knoevenagel condensation was subjected to Michael addition reaction by applying the appropriate 1,3-dinucleophiles. Dimedone, 4-hydroxycoumarin, 1,3-cyclohexanedione, 4-hydroxy-6-methyl-pyrone were used as Michael donors. 1 mmol of each Knoevenagel products reacted with 1.2 mmol of dinucleophile to afford the final products. The reactions were refluxed using ethanol as solvent and catalytic amount of piperidine (20 mol%). The progression of reaction was monitored using TLC with v:v/1:1 ratio of EtOAc/*n*-hexane as the solvent eluent. The final products were recrystallized from ethanol or isopropyl alcohol to provide purified products.

### Spectral data for all novel synthesized compounds

The aldehydes and Knoevenagel products (compounds **3a,b** and compounds **5a–d** except **5c**) were characterized in our previous studies^50,51^.

#### Methyl (E)-2-cyano-4-(4-(2-(piperidin-1-yl)ethoxy)phenyl)but-3-enoate (compound **5c**)

Bright yellowish white powder; yield = 73%; m.p.:189 °C; FT-IR (KBr): ῡ (cm^−1^) = 600, 763, 831, 1092, 1187, 1258, 1323, 1435, 1590, 1710, 2223, 2459, 2853, 2948, 3032; H NMR (DMSO-d_6_, 300 MHz): δ (ppm) = 1.58 (brs, CH_2_, 1H), 1.82 (brs, 2CH_2_, 4H), 3.07 (brs, 2H, NCH_2_), 3.47 (brs, CH_2_NCH_2_, 4H), 3.86 (s, OCH_3_, 3H), 4.60 (brs, OCH_2_, 2H), 7.22 (d, J = 9 Hz, CH_Ar_, 2H), 8.11 (d, J = 9 Hz, CH_Ar_, 2H), 8.35 (s, CH, 1H); ^13^C NMR (DMSO-d_6_, 75 MHz): δ (ppm) = 21.7, 22.8, 53.0, 53.6, 54.9, 63.4, 99.2, 116.0, 116.6, 124.9, 133.9, 154.9, 162.2, 163.2. HRMS (APCI-TOF) calcd. for C_18_H_22_N_2_O_3_: m/z = 314.1630, Found: 314.1623 (M + H)^+^.

#### 2-Amino-5-oxo-4-(4-(2-(piperidin-1-yl)ethoxy)phenyl)-5,6,7,8-tetrahydro-4H-chromene-3-carbonitrile (compound **7a**)

Yellowish powder; yield = 59%; m.p.: 131–133 °C; FT-IR (KBr): ῡ (cm^−1^) = 536, 953, 1069, 1163, 1209, 1239, 1364, 1510, 1601, 1683, 2189, 2546, 2637, 2944, 2963, 3158, 3380; H NMR (DMSO-d_6_, 300 MHz): δ (ppm) = 1.50 (brs, CH_2_, 2H), 1.74–1.77 (m, 2CH_2_, 4H), 1.84–2.00 (m, CH_2_, 2H), 2.21–2.32 (m, CH_2_, 2H), 2.56–2.63 (m, CH_2_, 2H), 3.07 (brs, CH_2_NCH_2_, 4H), 3.28 (t, *J* = 6 Hz, NCH_2_, 2H), 4.16 (s, CH, 1H), 4.35 (t, *J* = 6 Hz, OCH_2_, 2H), 6.91 (d, *J* = 9 Hz, CH_Ar_, 2H), 7.00 (s, NH_2_, 2H), 7.10 (d, *J* = 9 Hz, CH_Ar_, 2H); ^13^C NMR (DMSO-d_6_, 75 MHz): δ (ppm) = 20.3, 22.1, 23.3, 26.9, 35.1, 36.8, 53.2, 55.5, 58.7, 63.3, 114.4, 114.8, 120.3, 128.7, 138.0, 156.8, 158.9, 164.6, 196.3. HRMS (APCI-TOF) calcd. for C_23_H_27_N_3_O_3_: m/z = 393.2052, Found: 393.2005 (M + H)^+^.

#### 2-Amino-4-(4-(2-morpholinoethoxy)phenyl)-5-oxo-5,6,7,8-tetrahydro-4H-chromene-3-carbonitrile (compound **7b**)

Amber crystals; yield = 67%; m.p.: 158–160 °C; FT-IR (KBr): ῡ (cm^−1^) = 537, 1001, 1111, 1257, 1366, 1509, 1612, 1669, 1687, 2193, 2875, 2948, 3142, 3298; ^1^H NMR (DMSO-d_6_, 300 MHz): δ (ppm) = 1.84–2.01 (m, CH_2_, 2H), 2.21–2.34 (m, CH_2_, 2H), 2.48 (t, *J* = 6 Hz, CH_2_NCH_2_, 4H), 2.60–2.63(m, CH_2_, 2H), 2.69 (t, *J* = 6 Hz, NCH_2_, 2H), 3.59 (t, *J* = 6 Hz, CH_2_OCH_2_, 4H), 4.06 (t, *J* = 6 Hz, OCH_2_, 2H), 4.15 (s, CH, 1H), 6.78 (d, *J* = 9 Hz, CH_Ar_, 2H), 6.99 (s, NH_2_, 2H), 7.08 (d, *J* = 9 Hz, CH_Ar_, 2H); ^13^C NMR (DMSO-d_6_, 75 MHz): δ (ppm) = 20.2, 26.9, 35.1, 36.8, 54.0, 57.5, 58.8, 65.6, 66.6, 114.5, 114.6, 120.3, 128.6, 137.4, 157.6, 158.8, 164.5, 196.3. HRMS (APCI-TOF) calcd. for C_22_H_25_N_3_O_4_: m/z = 395.1845, Found: 395.1839 (M + H)^+^.

#### 2-Amino-7,7-dimethyl-5-oxo-4-(4-(2-(piperidin-1-yl)ethoxy)phenyl)-5,6,7,8-tetrahydro-4H-chromene-3-carbonitrile (compound **7c**)

Light yellow powder; yield = 63%; m.p.: 165–167 °C; FT-IR (KBr): ῡ (cm^−1^) = 559, 840, 1042, 1142, 1217, 1255, 1362, 1510, 1607, 1683, 2189, 2547, 2635, 2939, 2959, 3151, 3371; ^1^H NMR (DMSO-d_6_, 300 MHz): δ (ppm) = 0.96 (s, CH_3_, 3H), 1.04 (s, CH_3_, 3H), 1.51 (brs, CH_2_, 2H), 1.75–1.78 (m, 2CH_2_, 4H), 2.10 (d, *J* = 18 Hz, CH, 1H), 2.26 (d, *J* = 18 Hz, CH, 1H), 2.45–2.58 (m, CH_2_, 2H), 3.12 (brs, CH_2_NCH_2_, 4H), 3.32 (brs, NCH_2_, 2H), 4.14 (s, CH, 1H), 4.36 (t, *J* = 6 Hz, OCH_2_, 2H), 6.90 (d, *J* = 9 Hz, CH_Ar_, 2H), 7.01 (s, NH_2_, 2H), 7.08 (d, *J* = 9 Hz, CH_Ar_, 2H); ^13^C NMR (DMSO-d_6_, 75 MHz): δ (ppm) = 22.0, 23.1, 27.2, 28.8, 32.2, 35.2, 50.4, 53.1, 55.4, 56.4, 63.1, 113.3, 114.8, 120.2, 128.8, 138.0, 156.7, 158.8, 162.6, 196.1. HRMS (APCI-TOF) calcd. for C_25_H_31_N_3_O_3_: m/z = 421.2365, Found: 421.2357 (M + H)^+^.

#### 2-Amino-7,7-dimethyl-4-(4-(2-morpholinoethoxy)phenyl)-5-oxo-5,6,7,8-tetrahydro-4H-chromene-3-carbonitrile (compound **7d**)

Lemon crystals; yield = 70%; m.p.: 247–248 °C; FT-IR (KBr): ῡ (cm^−1^) = 606, 848, 1042, 1133, 1211, 1245, 1372, 1459, 1508, 1606, 1649, 2193, 2703, 2750, 2882, 2960, 3080, 3366, 3563; ^1^H NMR (DMSO-d_6_, 300 MHz): δ (ppm) = 0.97 (s, CH_3_, 3 H), 1.06 (s, CH_3_, 3 H), 2.10 (d, *J* = 15 Hz, CH, 1 H), 2.27 (d, *J* = 15 Hz, CH, 1 H), 2.52 (m, CH_2_, 2 H), 3.23 (brs, NCH_2_, 2 H), 3.50 (brs, CH_2_NCH_2_, 4 H), 3.79 (brs, CH_2_O, 2 H), 3.95 (brs, CH_2_O, 2 H), 4.16 (s, CH, 1 H), 4.38 (brs, OCH_2_, 2 H), 6.94 (d, *J* = 9 Hz, CH_Ar_, 2 H), 6.99 (s, NH_2_, 2 H), 7.11 (d, *J* = 9 Hz, CH_Ar_, 2 H); ^13^C NMR (DMSO-d_6_, 75 MHz): δ (ppm) = 27.2, 28.9, 32.2, 35.2, 50.4, 52.1, 55.4, 58.9, 62.5, 63.7, 113.3, 114.9, 120.2, 128,8, 138,2, 156.6, 158.8, 162.7, 196.1. HRMS (APCI-TOF) calcd. for C_24_H_29_N_3_O_4_: m/z = 423.2158, Found: 423.2151 (M + H)^+^.

#### 2-Amino-7-methyl-5-oxo-4-(4-(2-(piperidin-1-yl)ethoxy)phenyl)-4H,5H-pyrano[4,3-b]pyran-3-carbonitrile (compound **7e**)

Orange powder; yield = 65%; m.p. 196–199 °C; FT-IR (KBr): ῡ (cm^−1^) = 544, 985, 1044, 1140, 1179, 1256, 1382, 1445, 1508, 1612, 1672, 1696, 2200, 2541, 2642, 2948, 3192, 3376, 3495; ^1^H NMR (DMSO-d_6_, 300 MHz): δ (ppm) = 1.52 (brs, CH_2_, 2H), 1.79 (t, *J* = 6.0 Hz, 2CH_2_, 4H), 2.23 (s, CH_3_, 3H), 3.15 (brs, CH_2_NCH_2_, 4H), 3.36 (brs, NCH_2_, 2H), 4.25 (s, CH, 1H), 4.39 (t, *J* = 6.0 Hz, OCH_2_, 2H), 6.30 (s, CH_sp2_, 1H), 6.93 (d, *J* = 9.0 Hz, CH_Ar_, 2H), 7.13 (d, *J* = 9.0 Hz, CH_Ar_, 2H), 7.22 (s, NH_2_, 2H); ^13^C NMR (DMSO-d_6_, 75 MHz): δ (ppm) = 19.7, 21.9, 23.0, 35.9, 53.1, 55.3, 58.4, 63.0, 98.4, 101.3, 114.9, 119.8, 129.1, 136.8, 157.0, 158.4, 158.5, 161.8, 163.2. HRMS (APCI-TOF) calcd. for C_23_H_25_N_3_O_4_: m/z = 407.1845, Found: 407.1786 (M + H)^+^.

#### 2-Amino-7-methyl-4-(4-(2-morpholinoethoxy)phenyl)-5-oxo-4H,5H-pyrano[4,3-b]pyran-3-carbonitrile (compound **7f**)

Reddish orange powder; yield = 68%; m.p. 195–196 °C; FT-IR (KBr): ῡ (cm^−1^) = 563, 945, 1042, 1133, 1248, 1372, 1459, 1508, 1606, 1649, 1685, 2193, 2499, 2626, 2960, 3193, 3366, 3563; ^1^H NMR (DMSO-d_6_, 300 MHz): δ (ppm) = 2.22 (s, CH_3_, 3H), 2.50 (brs, CH_2_NCH_2_, 4H), 2.70 (t, *J* = 6.0 Hz, NCH_2_, 2H), 3.59 (t, *J* = 6.0 Hz, CH_2_OCH_2_, 4H), 4.06 (t, *J* = 6.0 Hz, OCH_2_, 2H), 4.24 (s, CH, 1 H), 6.26 (s, CH_sp2_, 1H), 6.88 (d, *J* = 9.0 Hz, CH_Ar_, 2H), 7.10 (d, *J* = 9.0 Hz, CH_Ar_, 2H), 7.19 (s, NH_2_, 2H); ^13^C NMR (DMSO-d_6_, 75 MHz): δ (ppm) = 19.7, 35.9, 54.0, 57.4, 58.5, 65.6, 66.5, 98.4, 101.4, 114.7, 119.8, 129.0, 136.2, 157.8, 158.3, 158.4, 161.8, 163.2. HRMS (APCI-TOF) calcd. for C_22_H_23_N_3_O_5_: m/z = 409.1637, Found: 409.1624 (M + H)^+^.

#### 2-Amino-5-oxo-4-(4-(2-(piperidin-1-yl)ethoxy)phenyl)-4H,5H-pyrano[3,2-c]chromene-3-carbonitrile (compound **7g**)

Light yellow crystals; yield = 63%; m.p: 185–186 °C; FT-IR (KBr): ῡ (cm^−1^) = 766, 1048, 1113, 1174, 1242, 1377, 1606, 1677, 1721, 2192, 2954, 3153, 3349; ^1^H NMR (DMSO-d_6_, 300 MHz): δ (ppm) = 1.50–1.74 (m, 3CH_2_, 6H), 2.95 (brs, 3CH_2_, 6H), 4.33 (brs, OCH_2_, 2H), 4.41 (s, CH, 1H), 6.93 (d, *J* = 8.5 Hz, CH_Ar_, 2H), 7.20 (d, *J* = 8.5 Hz, CH_Ar_, 2H), 7.42–7.51 (m, CH_Ar_, NH_2_, 4H), 7.70–7.73 (m, CH_Ar_, 1H), 7.89–7.91 (m, CH_Ar_, 1H); ^13^C NMR (DMSO-d_6_, 75 MHz): δ (ppm) = 21.7, 22.9, 36.6, 53.1, 55.2, 58.5, 62.8, 104.6, 113.5, 115.1, 117.0, 119.8, 123.0, 125.2, 129.4, 133.4, 136.7, 152.6, 153.7, 157.1, 158.4, 160.0. HRMS (APCI-TOF) calcd. for C_26_H_25_N_3_O_4_: m/z = 443.1845, Found: 443.1833 (M + H)^+^.

#### 2-Amino-4-(4-(2-morpholinoethoxy)phenyl)-5-oxo-4H,5H-pyrano[3,2-c]chromene-3-carbonitrile (compound **7h**)

Light yellow crystals; yield = 71%; m.p: 191–193 °C; FT-IR (KBr): ῡ (cm^−1^) = 763, 1035, 1100, 1171, 1375, 1605, 1679, 1729, 2189, 2860, 2937, 3148, 3272, 3413; ^1^H NMR (DMSO-d_6_, 300 MHz): δ (ppm) = 2.46 (brs, 2CH_2_, 4H), 2.67 (brs, CH_2_, 2H), 3.56 (brs, 2CH_2_, 4H), 4.01 (t, *J* = 5.5 Hz, OCH_2_, 2H), 4.38 (s, CH, 1H), 6.86 (d, *J* = 8 Hz, CH_Ar_, 2H), 7.15 (d, *J* = 8 Hz, CH_Ar_, 2H), 7.38 (brs, NH_2_, 2H), 7.44–7.50 (m, CH_Ar_, 2H), 7.70 (t, *J* = 7.5 Hz, CH_Ar_, 1H), 7.94 (d, *J* = 7.5 Hz, CH_Ar_, 1H); ^13^C NMR (DMSO-d_6_, 75 MHz): δ (ppm) = 36.7, 53.9, 57.4, 58.9, 65.6, 66.5, 104.8, 113.5, 114.9, 117.0, 119.8, 122.9, 125.1, 129.2, 133.3, 135.9, 152.6, 153.6, 157.9, 158.4, 160.0. HRMS (APCI-TOF) calcd. for C_25_H_23_N_3_O_5_: m/z = 445.1637, Found: 445.1625 (M + H)^+^.

### *In vitro* assays

#### Modified Ellman’s method for cholinesterase inhibition assays

All the synthesized compounds were screened for their cholinesterase activity by Ellman’s spectrophotometric method with a slight modification^24^. All chemicals, reagents and reference drug, AChE (E.C.3.1.1.7, from electric eel), acetylthiocholine iodide (ATCI), BuChE (E.C.3.1.1.8, from equine serum), butyrylthiocholine iodide (BTCI), 5,5′-dithiobis-(2-nitrobenzoic acid) (DTNB) and donepezil hydrochloride) used in the procedure were supplied from Sigma-Aldrich and Fluka. In brief, enzymatic reactions were accomplished in 96-well plates with total volume of 270 µL. Each well contained 0.1 M phosphate buffer (pH 7.4), 0.08 U/mL AChE or 0.05 U/mL BuChE and 0.3 mM of DTNB. In order to considering the influence of DMSO percentage on the enzymatic activity of AChE, different concentrations of the test sample were prepared in final concentrations of 0.2% DMSO. Then contents were incubated for 15 min at room temperature. The reaction was started with the addition of 20 µL of 0.45 mM substrate (ATCI/BTCI) into premixed enzyme/buffer/inhibitor mixture and monitored by using plate reader Synergy HT, Biotek, USA at 412 nm. The activity in the absence of sample compounds was considered as a blank. The percentage inhibition was calculated from (blank – test sample)/blank × 100. IC_50_ values were calculated as concentration of the compound that produces 50% enzyme activity inhibition, using curve expert.

#### Kinetic study of AChE and BuChE inhibition

To determine the mechanisms of action of **5c** and **7c**, a series of experiments were performed at different concentrations of the substrate ATCI/BTCI. The inhibitor concentrations were (IC_50_, 2IC_50_). Substrates ATCI/BTCI concentrations were 0, 0.13, 0.3, 0.45, 0.675, 1.01 and 1.51 mM in all kinetic studies. Pre-incubation and measurement time were the same as reported in the perivous assay protocol. Kinetic characterization analysis was done spectrometrically at 412 nm.

#### DPPH antioxidant assay

The DPPH assay method is based on the reduction of 2,2-diphenyl-1-picrylhydrazyl or DPPH (a stable free radical). To 180 µL of 0.1 mM DPPH reagent (prepared in methanol), 20 µL of different concentrations of synthesized compounds up to 100 µM in methanol were added, mixed well, incubated at room temperature for 30 min. Quercetin was used as positive control. As the DPPH is getting reduced with an antioxidant it will change its color to yellow and this color changes are recorded using plate reader Synergy HT, Biotek, USA at 517 nm. The percentage of scavenging activity was calculated as IC_50_, in which a lower IC_50_ suggests a stronger antioxidant.

### Molecular modeling

All molecular modeling studies were carried out on a 64 bit Ubuntu (18.04) machine with Intel core i7–7700 CPU (3.60 GHz) and GeForce GTX 1050Ti graphic processor. The setup and the methodologies for computational studies is in accordance with the literature^52,53^. Using Glide XP precision, molecular docking was used for docking of the best *in vitro* compounds. Docking was employed by OPLS_2005 force-field through sampling flexible ligand structures as well as ring conformations and nitrogen inversions. Epik state penalties were added to the final scores and a post-docking minimization was performed to further improve the accuracy of the results. A set of 5000 poses were set for initial step of docking and 800 best poses for each ligand were submitted for energy minimization^54–56^. The binding for generation of the grid file was inferred from previous studies related to donepezil^57^. Protein structure of AChE and BuChE were extracted from Protein Data Bank (PDB ID: 4EY7 and 1P0I)^57,58^. For donepezil and AChE molecular dynamics, the same enantiomer were used as in 4EY7 structure. For donepezil and BuChE molecular dynamics, the enantiomer with more negative docking energy was subjected to molecular dynamics. The same approach was used for molecular dynamics of compound **7c** and BuChE. Also as our final products displayed enol-keto tautomerism, the tautomer with more negative docking score was utilized for further MD assessment.

To investigate the MD simulation of AChE with the best compounds determined in the previous steps, Desmond v5.3 (Schrodinger suite 2018–1) was utilized^59,60^. OPLS_2005 force-field was applied for the simulation, in a drenched box with SPC water model and a concentration of 0.15 M of sodium chloride. Steepest descent minimization was carried out following the Limited-memory Broyden-Fletcher-Goldfarb-Shanno (LBFGS) method of energy minimization to converge the system to a gradient of 1 kcal/mol/Å with a maximum iteration of 2000. MD simulation was settled in NPT ensemble (constant number of atoms, constant pressure i.e. 1.01325 bar and constant temperature i.e. 310 K) and before running the MD simulation the temperature of system was raised to 400 K to remove the non-selective interactions for 0.5 nanosecond and returned to the normal 310 K after that period (simulated annealing). The Nose-Hoover chain and Martyna-Tobias-Klein approach were used as the default thermostat and barostat with 1.0 ps and 2.0 ps interval by isotropic coupling style respectively. For computation of near and far range forces, a 2 fs and 6 fs Reversible Reference System Propagator Algorithm (RESPA) integrator time-step, was exploited. Summation of long-range electrostatic forces was performed by Particle Mesh Ewald (PME) method. A cut-off radius of 9.0 Å was set for Coulombic forces. SHAKE algorithm was used to impose constraint on the geometry of water molecules and heavy atom bond lengths with hydrogen and therefore speed-up the calculations with acceptable precision^61^.

The Root Mean Square Deviation (RMSD), Root Mean Square Fluctuation (RMSF) of both proteins and ligands and also ligands’ torsional profile were monitored throughout simulation (50 nanosecond) in reference to the first frame. Interactions lasting more than 30% of the time of simulation were documented in final results.

### Pharmacokinetics profile

In this study the SwissADME server was utilized to assess the computational properties of the best inhibitors of AChE and BuChE in comparison with donepezil^44^. Physicochemical, lipophilicity, druglikeness and leadlikeness and metabolism and other pharmacokinetics related properties were assessed. The admetSAR was also utilized for further consideration of pharmacokinetics profile and assessment of other ADME properties which were not provided by SwissADME^45^.

## Supplementary information

Supplementary Information
