# Supplementary material for: Phenoxyethyl Piperidine/Morpholine Derivatives as PAS and CAS Inhibitors of Cholinesterases: Insights for Future Drug Design
Source: Sci Rep. 2019 Dec 27;9:19855. doi: 10.1038/s41598-019-56463-2 (PMC6934599; doi:10.1038/s41598-019-56463-2)
Supplement: Supplementary file 1 — Supplementary Information [file 41598_2019_56463_MOESM1_ESM.doc]

**Phenoxyethyl Piperidine/Morpholine Derivatives as PAS and CAS Inhibitors of Cholinesterases: Insights for Future Drug Design**

Yaghoub Pourshojaei,^a,b^ Ardavan Abiri,^a^ Khalil Eskandari,*^a^ Zahra Haghighijoo,^c^ Najmeh Edraki,*^,c^ Ali Asadipour,^a^

*^a^Department of Medicinal Chemistry, Faculty of Pharmacy and Pharmaceutics Research Center, Kerman University of Medical Sciences, Kerman, Iran; ^b^Neuroscience Research Center, Institute of Neuropharmacology, Kerman University of Medical Sciences, Kerman, Iran; ^c^Medicinal and Natural Products Chemistry Research Center, Shiraz University of Medical Sciences, Shiraz, Iran; Corresponding authors:* [*khalileskandari@yahoo.com*](mailto:khalileskandari@yahoo.com)*;* [*najmeh_edraki@yahoo.com*](mailto:najmeh_edraki@yahoo.com)

**Graphical abstract**

**
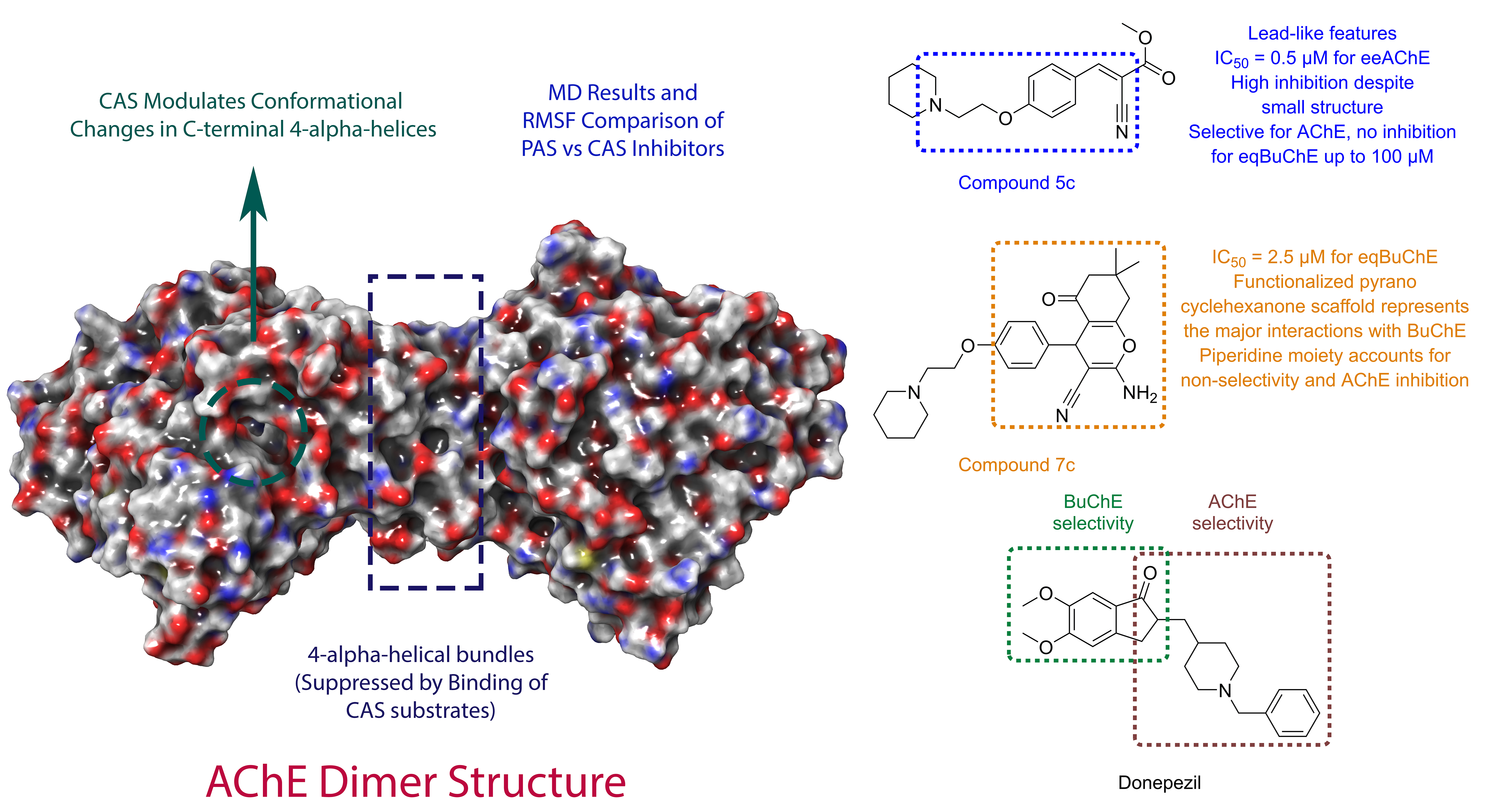
**

**
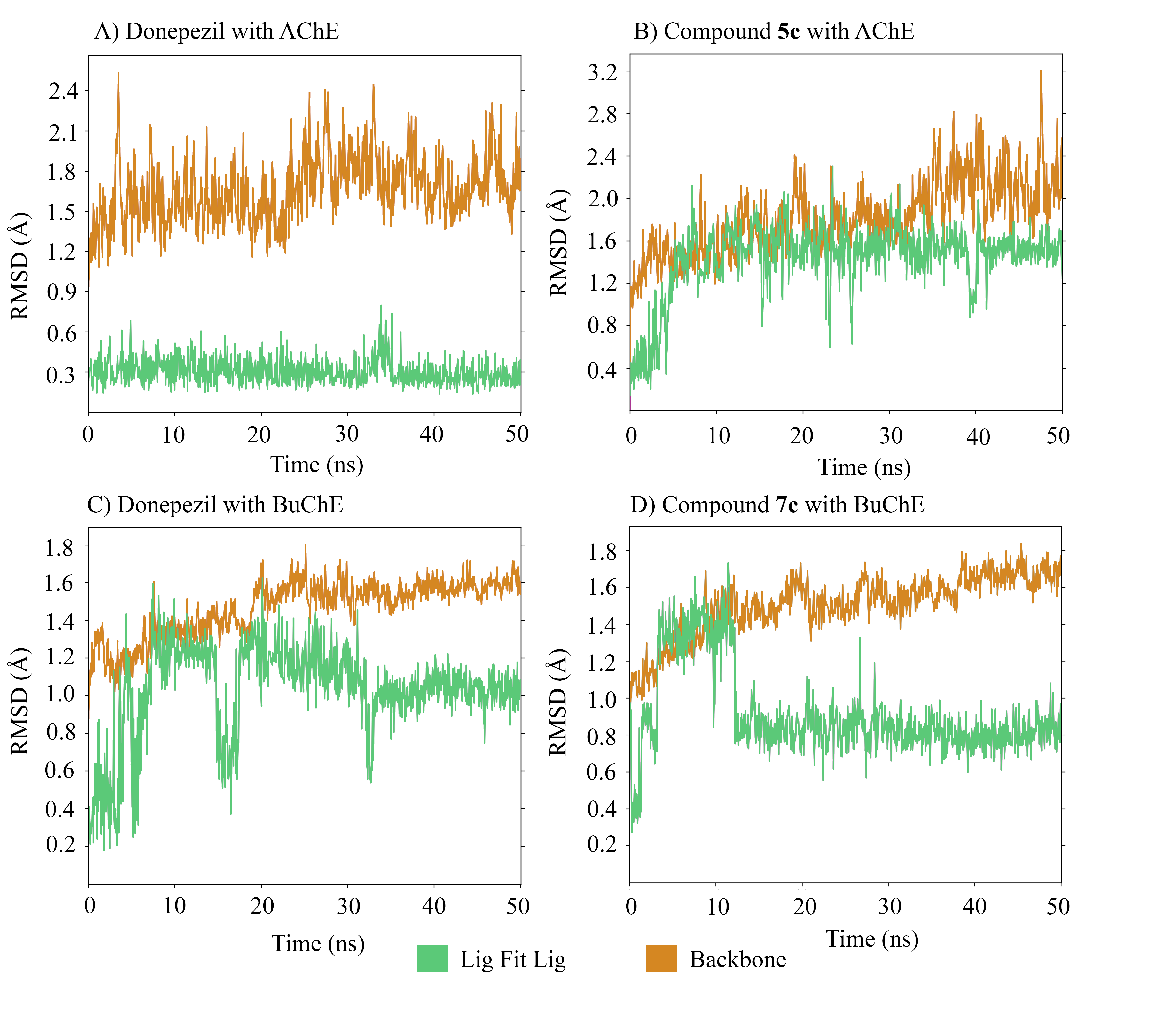
**

**Fig. S1.** RMSD values for the hAChE (A and B) and hBuChE (C and D).


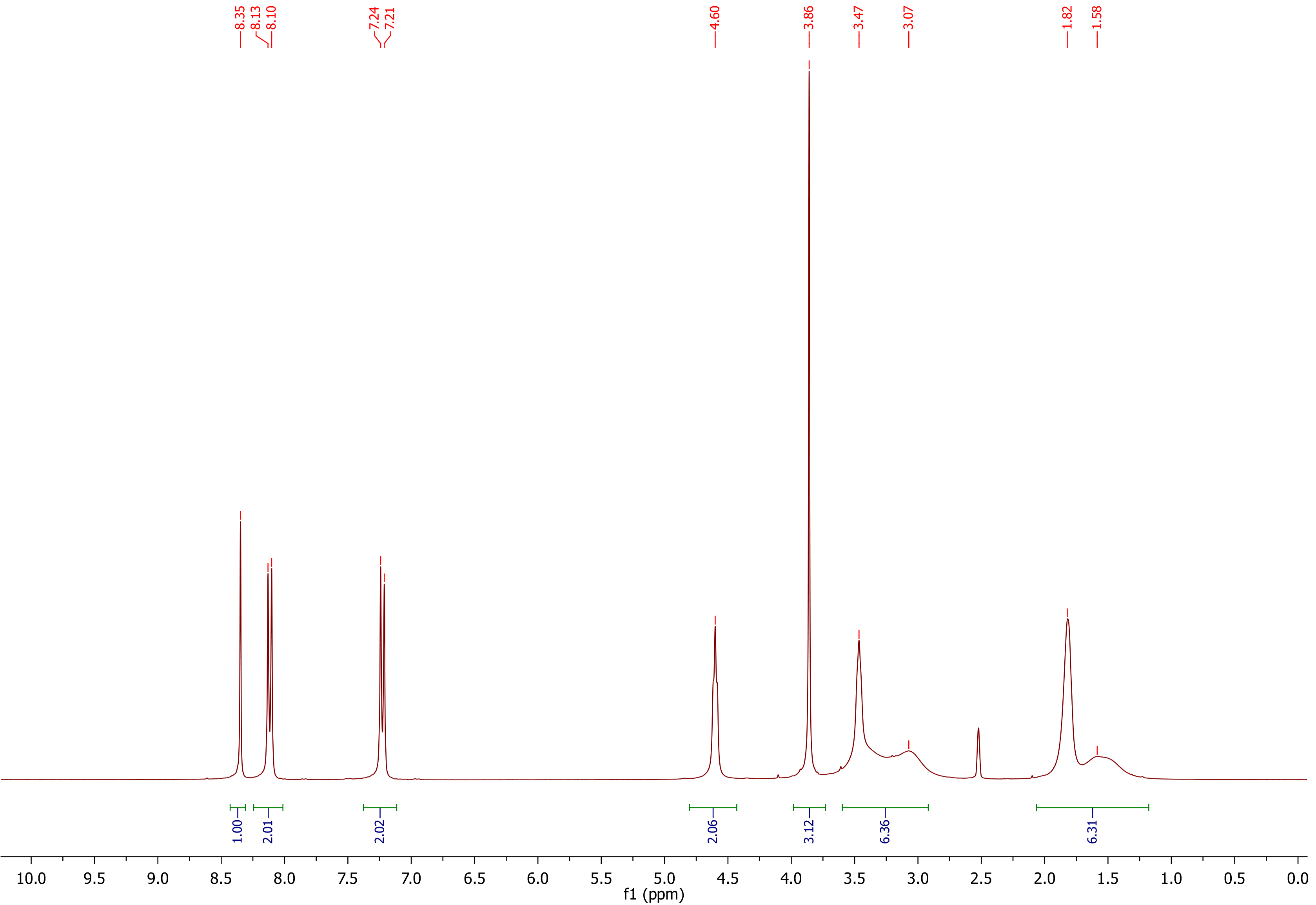


**Fig. S2.** ^1^H NMR spectrum of product **5c**


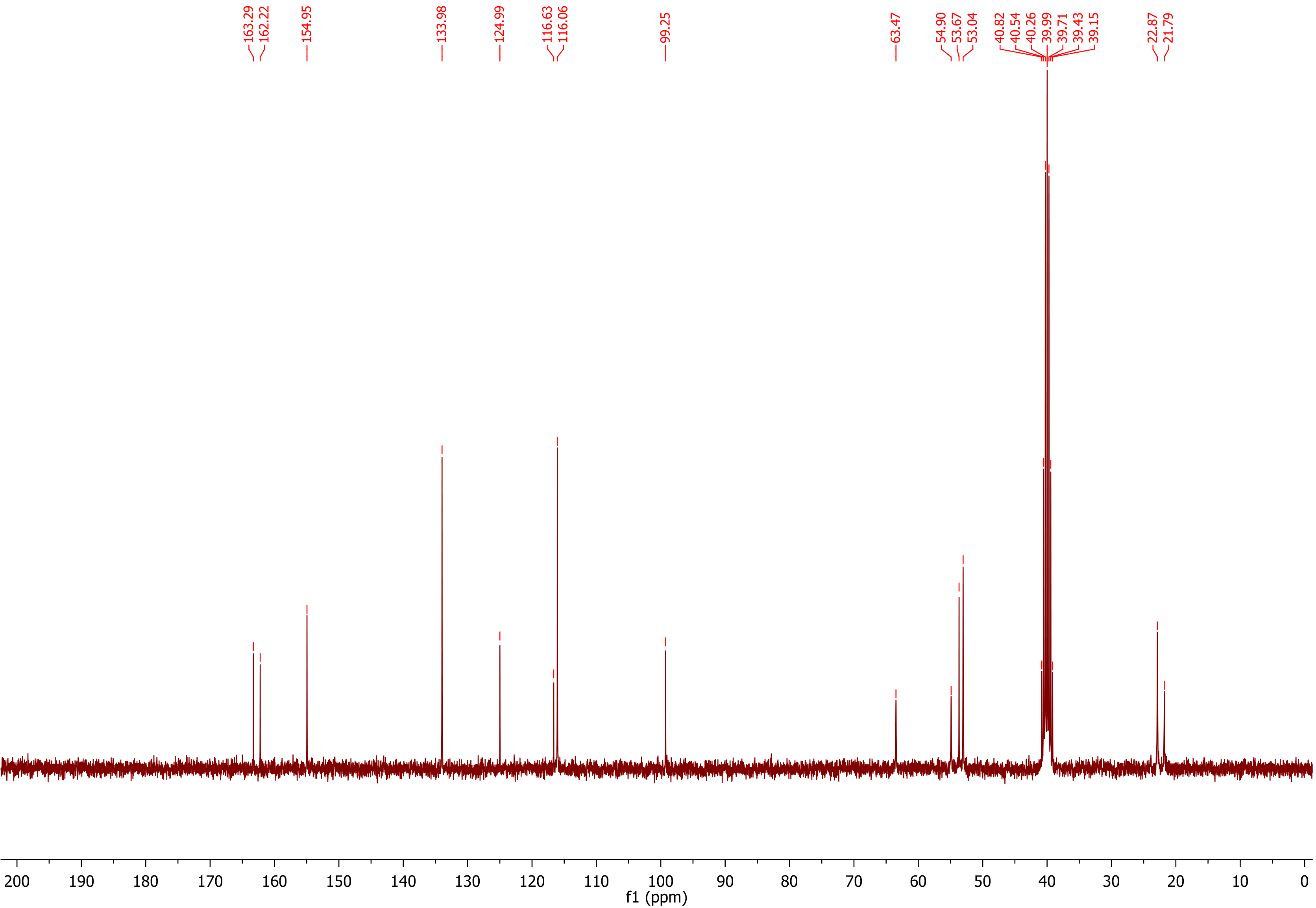


**Fig. S3.** ^13^C NMR spectrum of product **5c**


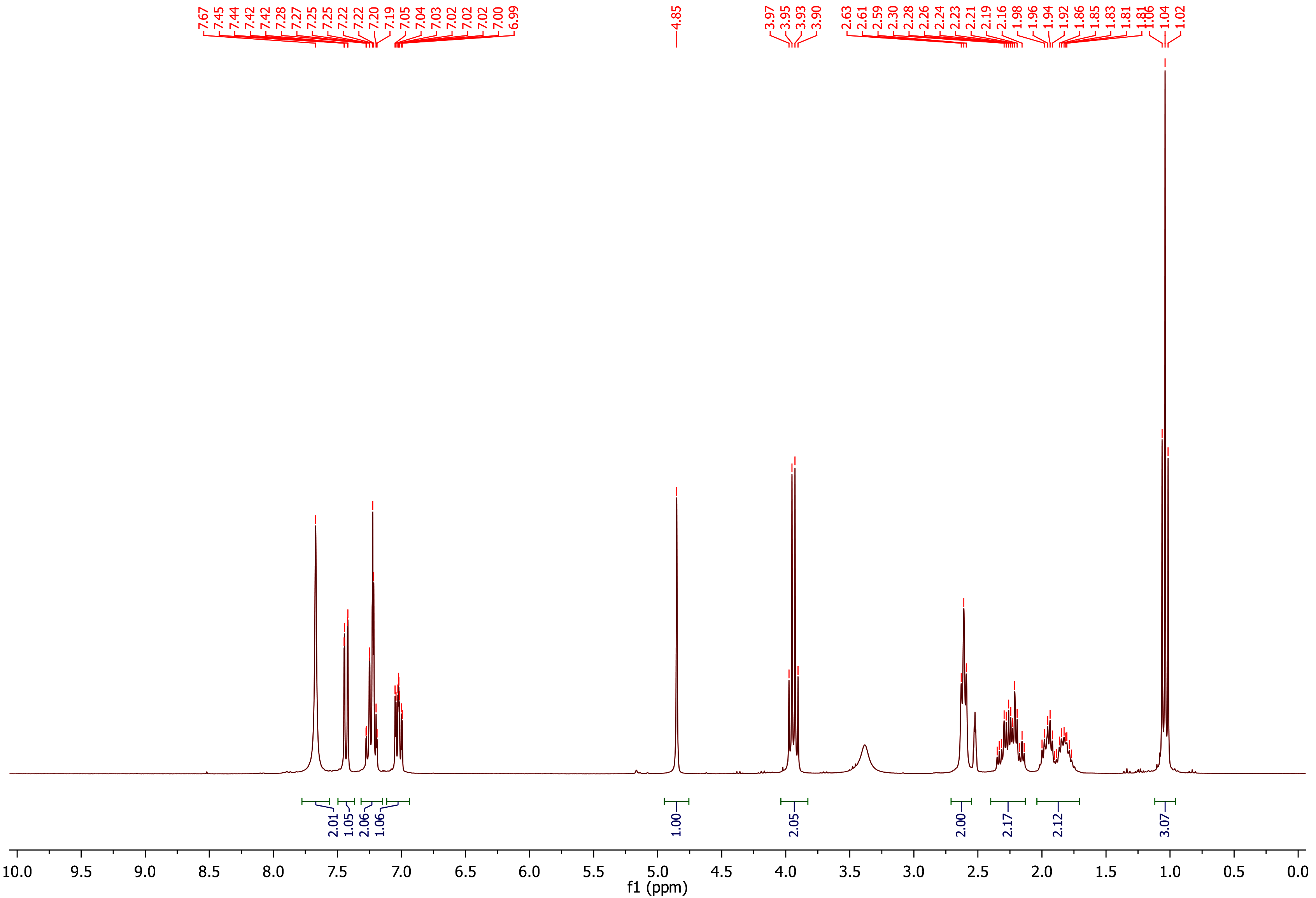


**Fig. S4.** ^1^H NMR spectrum of product **7a**


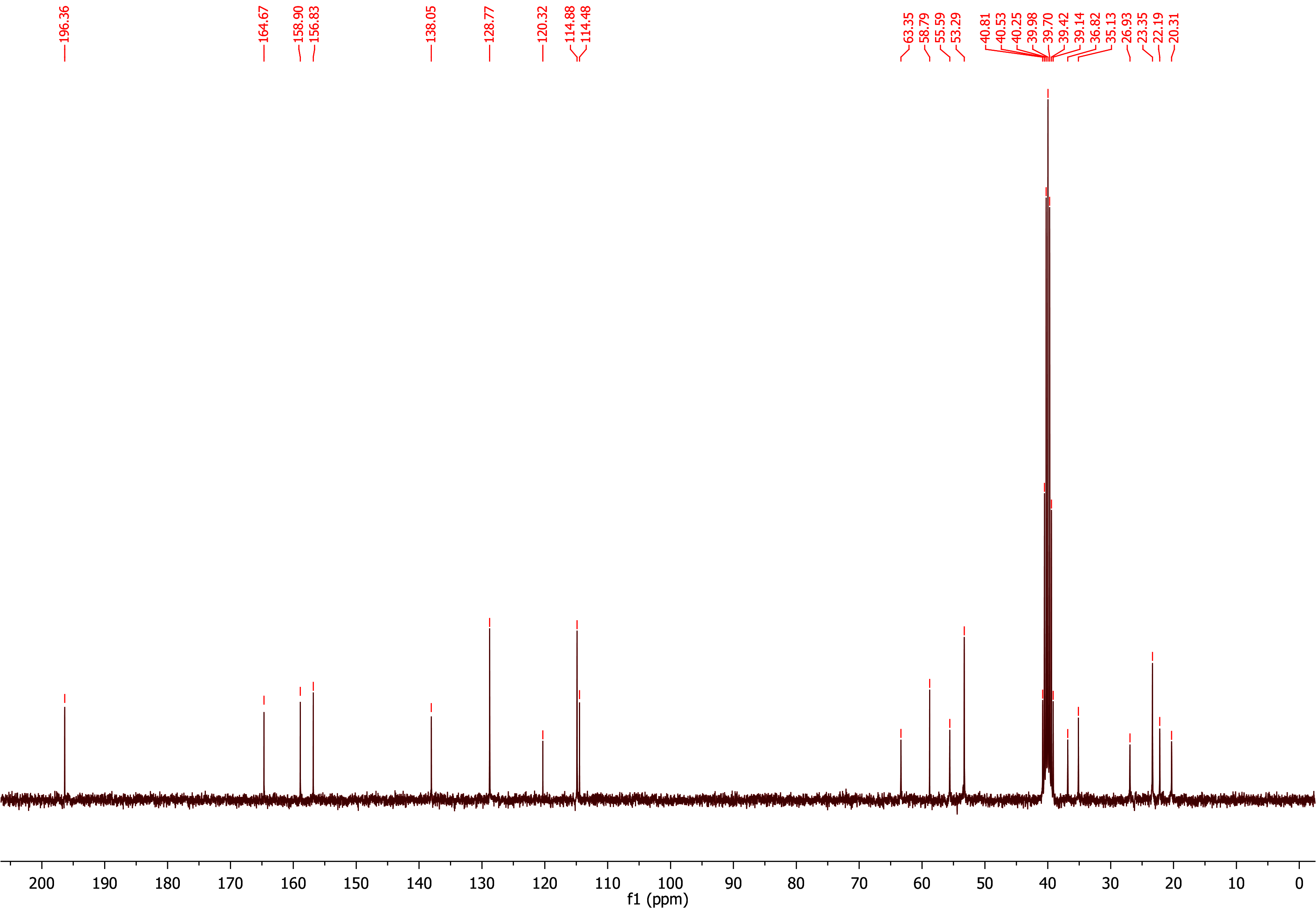


**Fig. S5.** ^13^C NMR spectrum of product **7a**


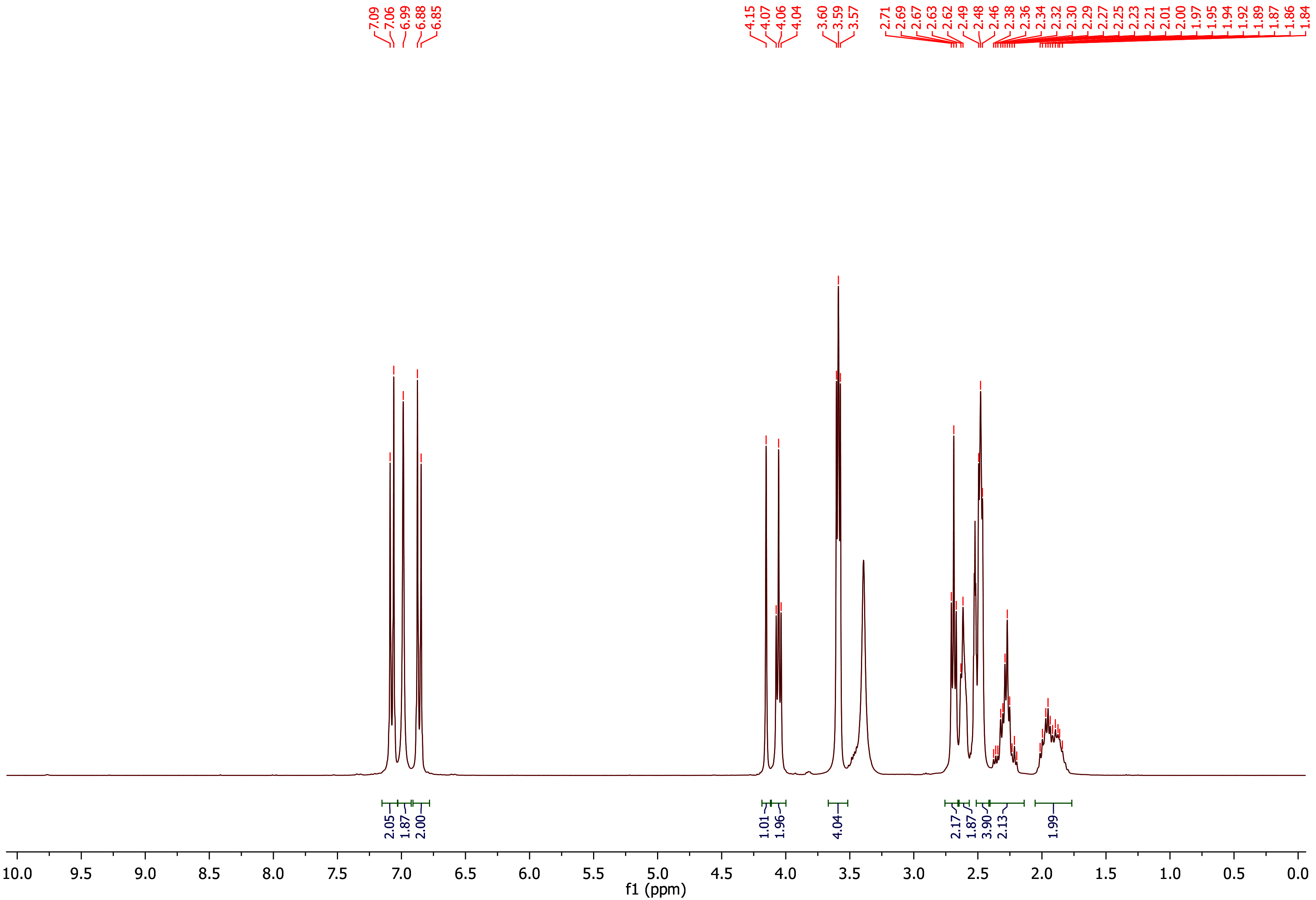


**Fig. S6.** ^1^H NMR spectrum of product **7b**


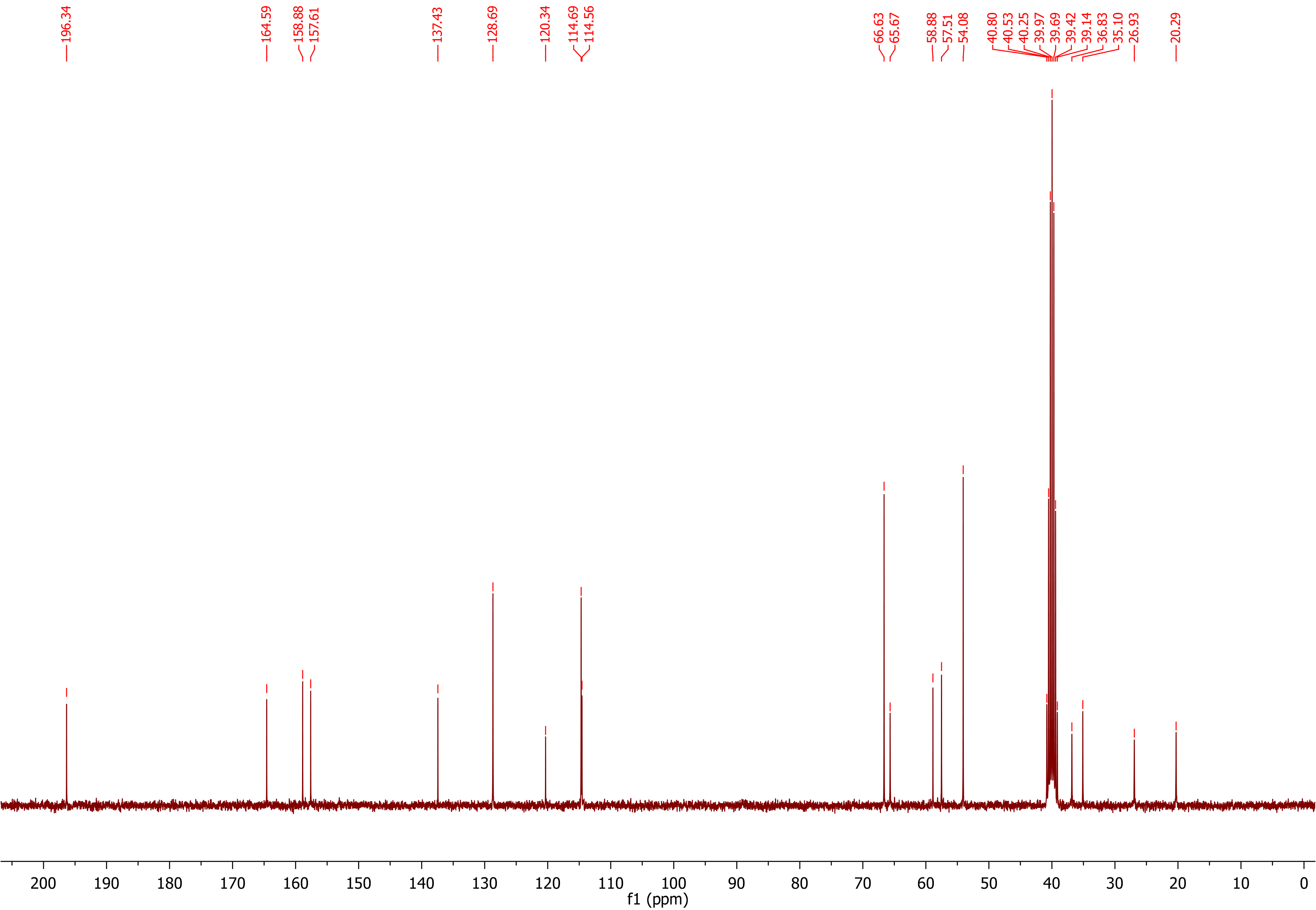


**Fig. S7.** ^13^C NMR spectrum of product **7b**

#
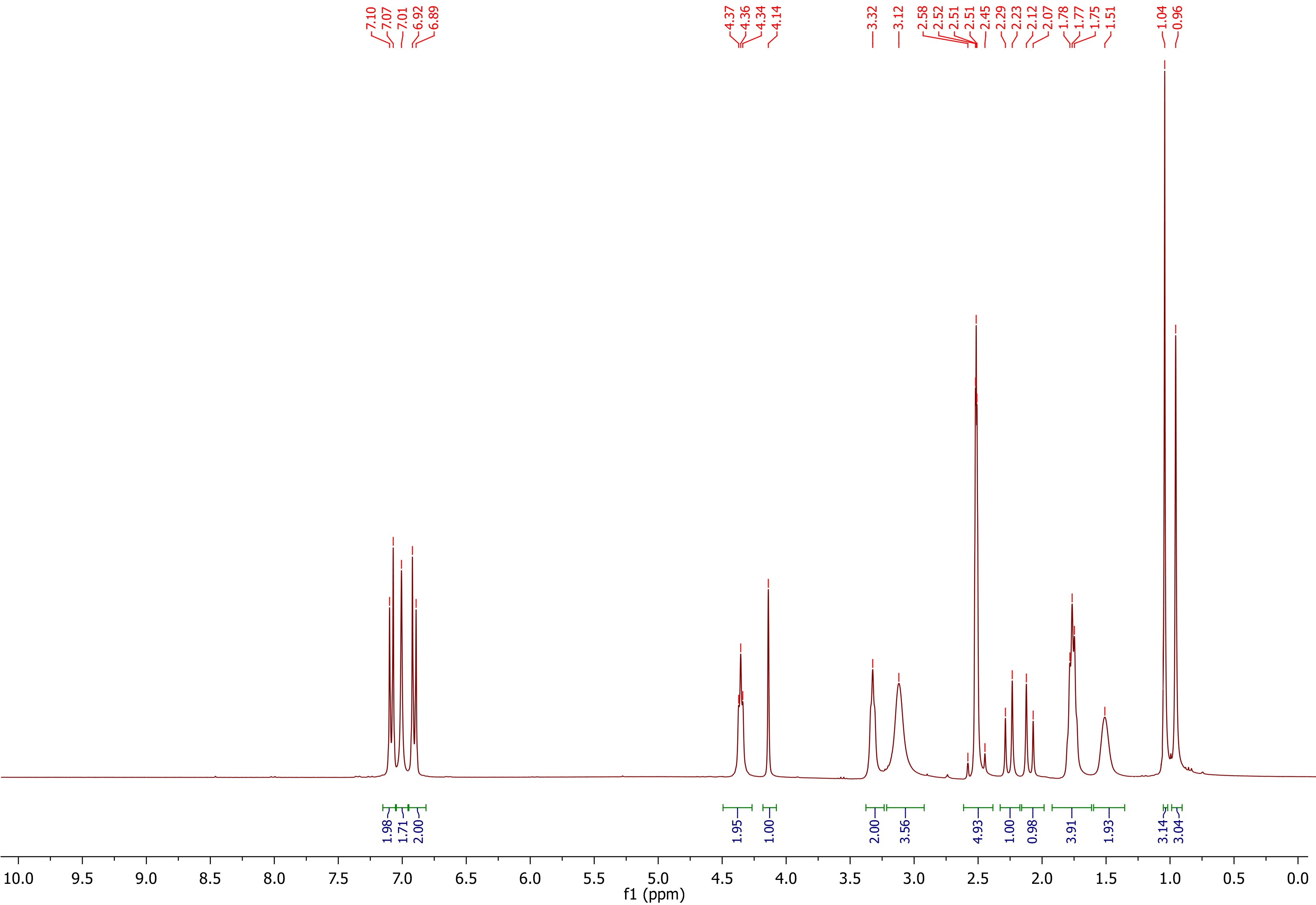


**Fig. S8.** ^1^H NMR spectrum of product **7c**

#
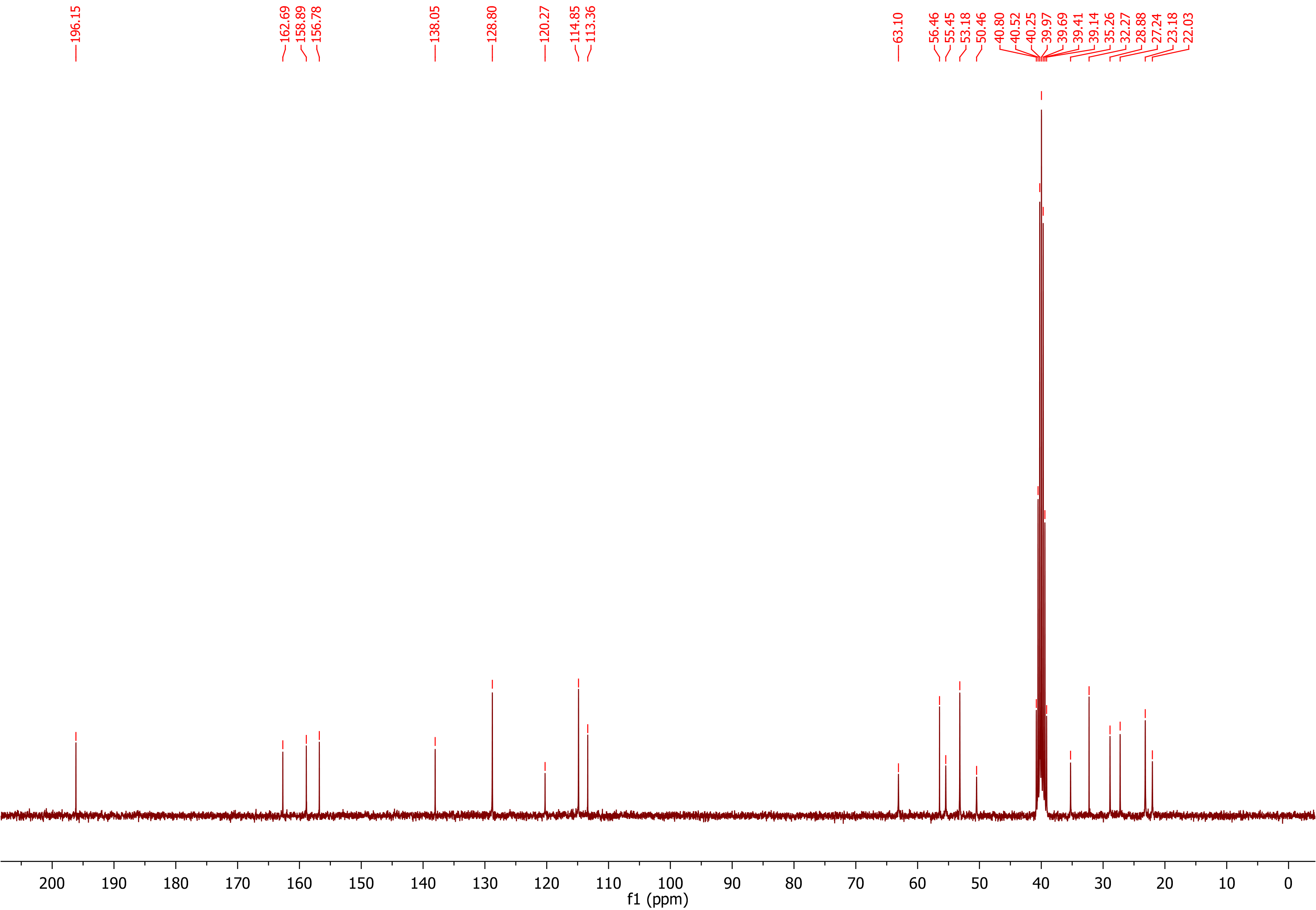


**Fig. S9.** ^13^C NMR spectrum of product **7c**

#
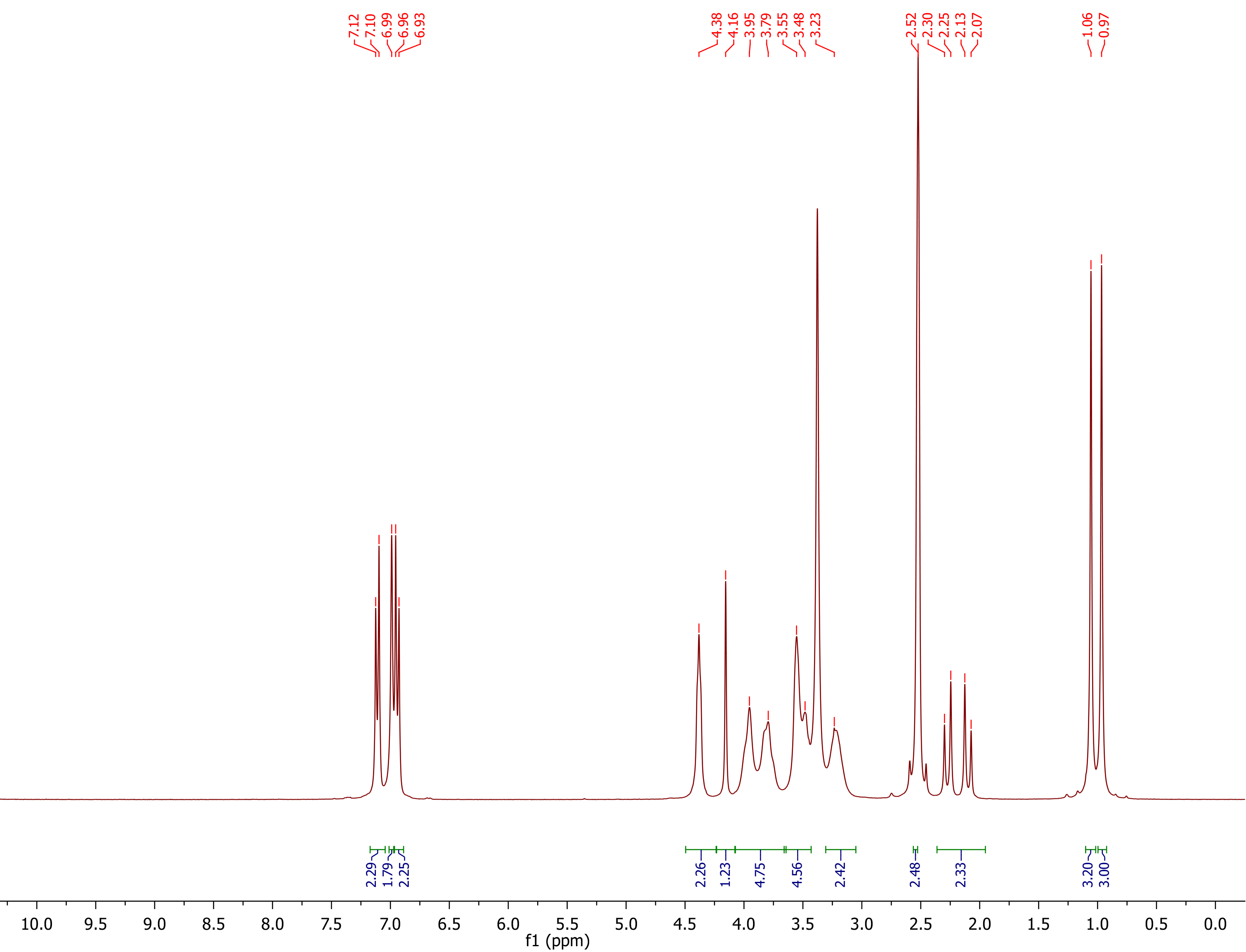


**Fig. S10.** ^1^H NMR spectrum of product **7d**

#
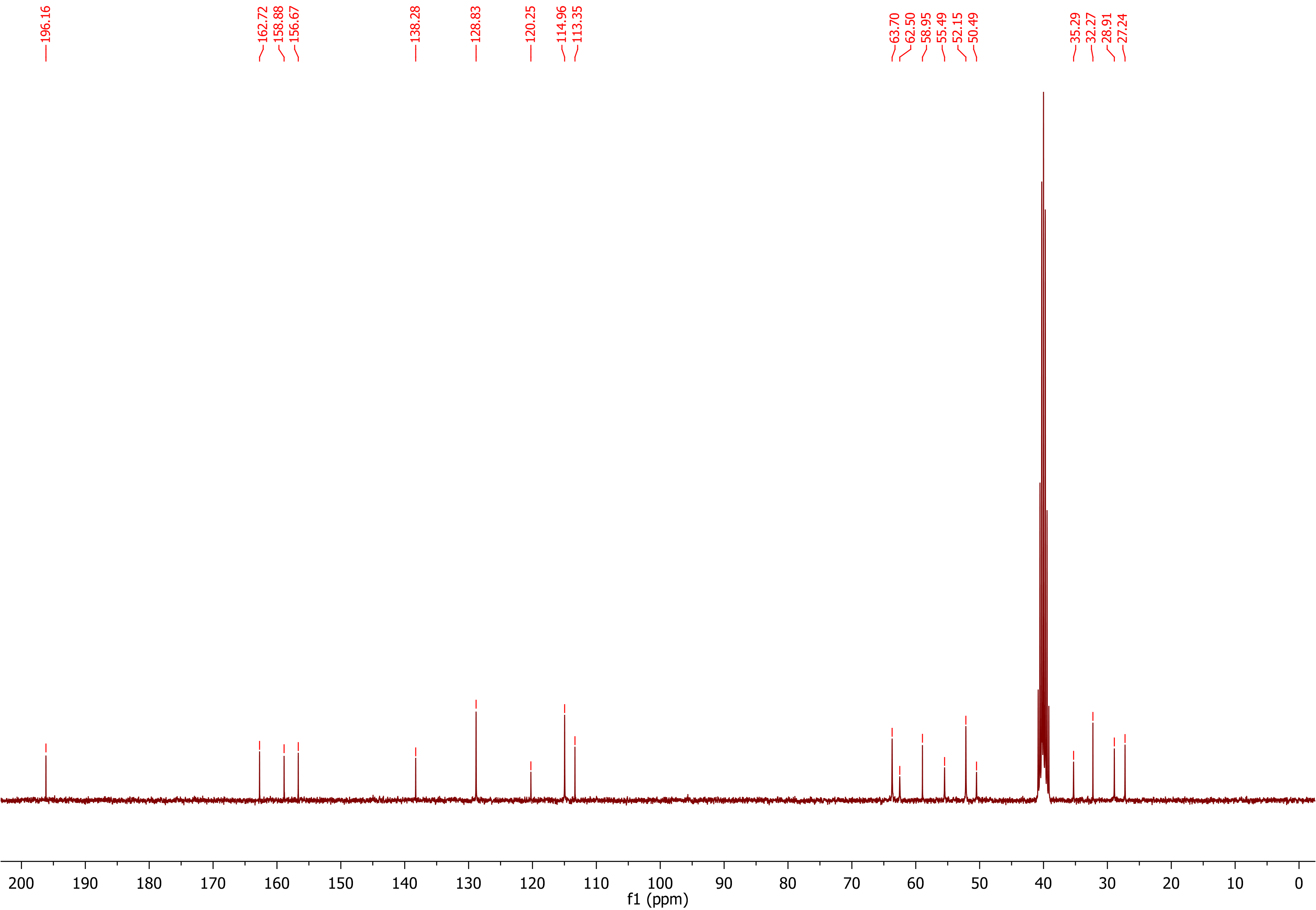


**Fig. S11.** ^13^C NMR spectrum of product **7d**

#
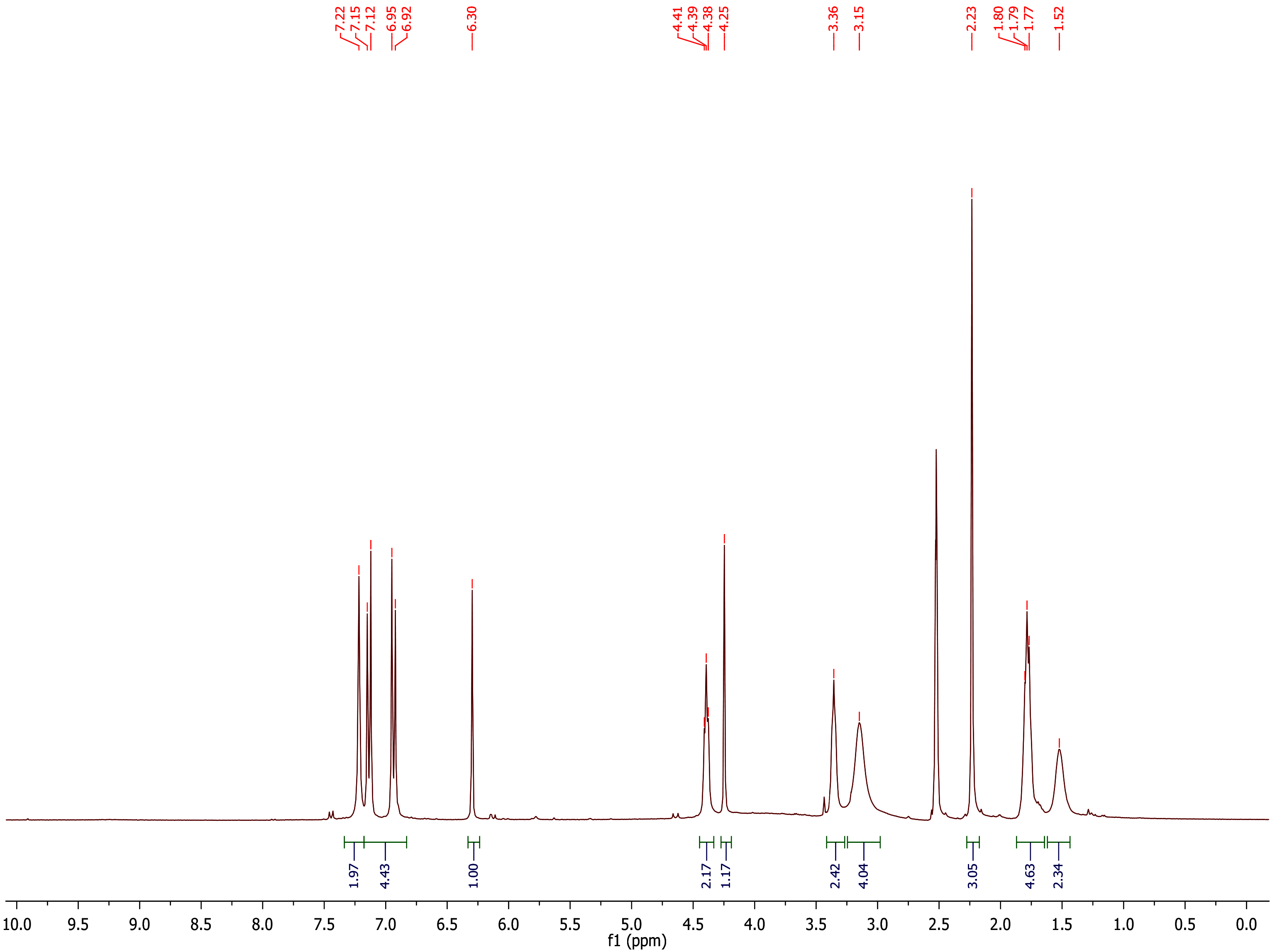


**Fig. S12.** ^1^H NMR spectrum of product **7e**

#
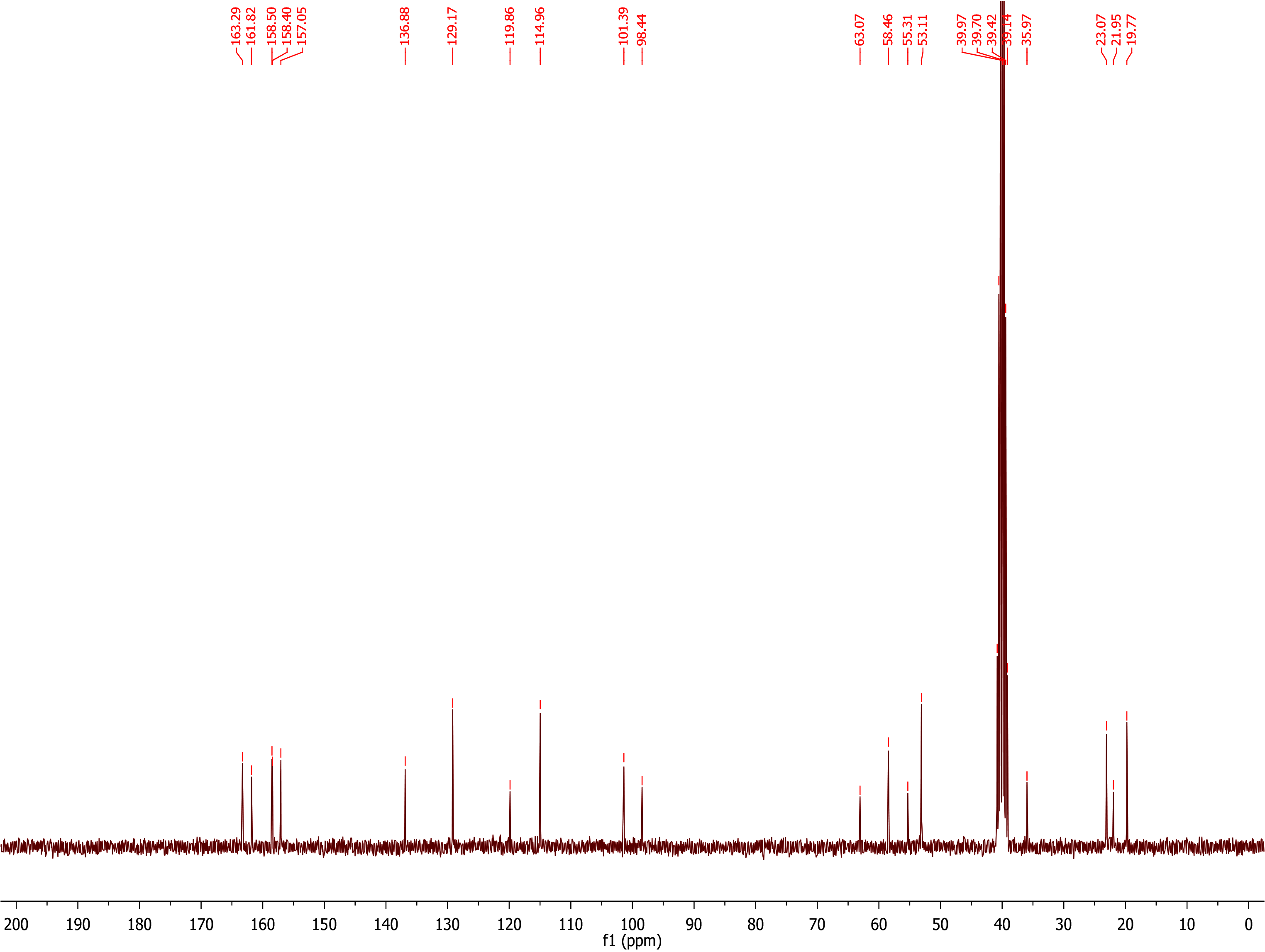


**Fig. 13.** ^13^C NMR spectrum of product **7e**


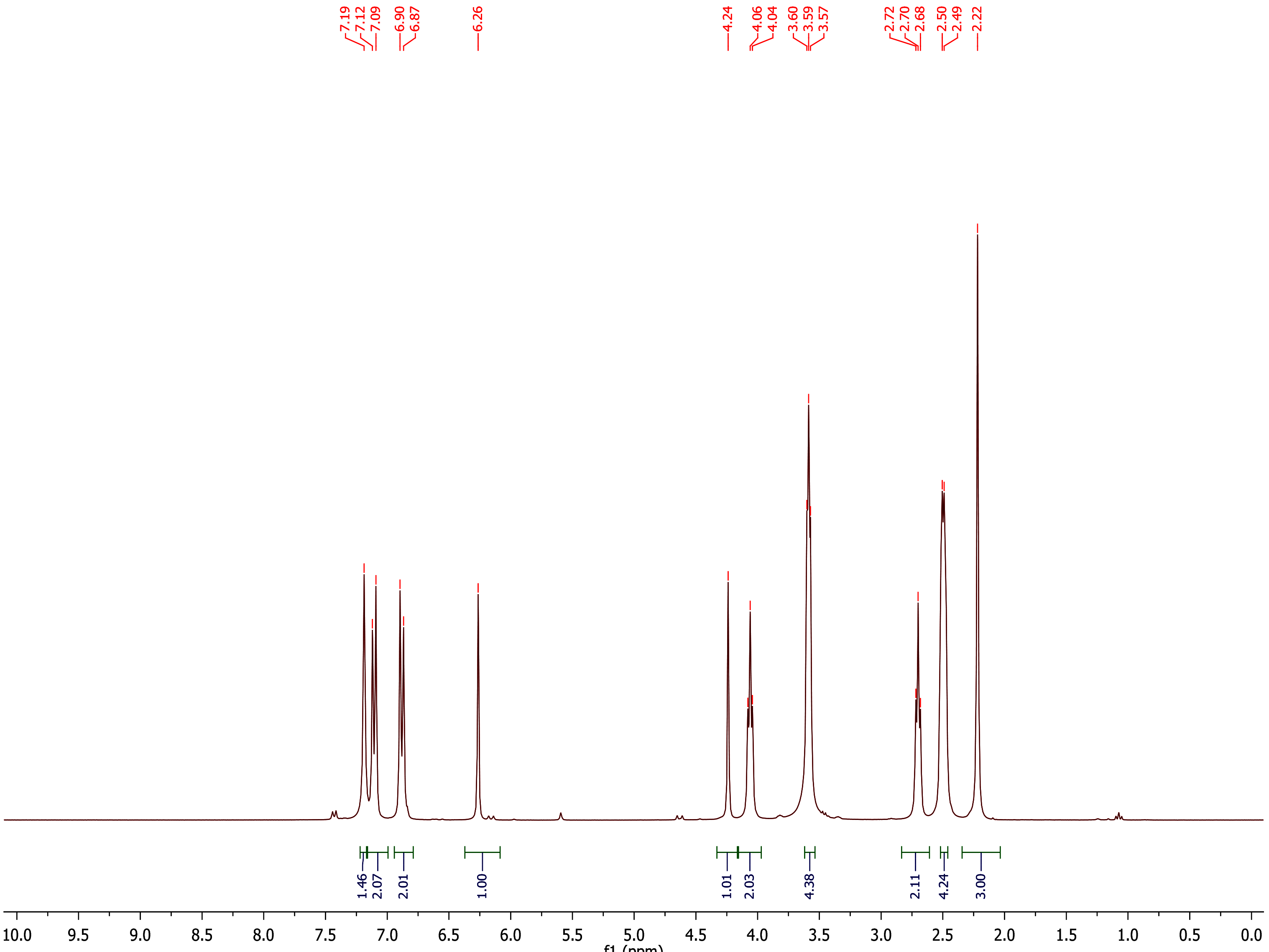


**Fig. S14.** ^1^H NMR spectrum of product **7f**


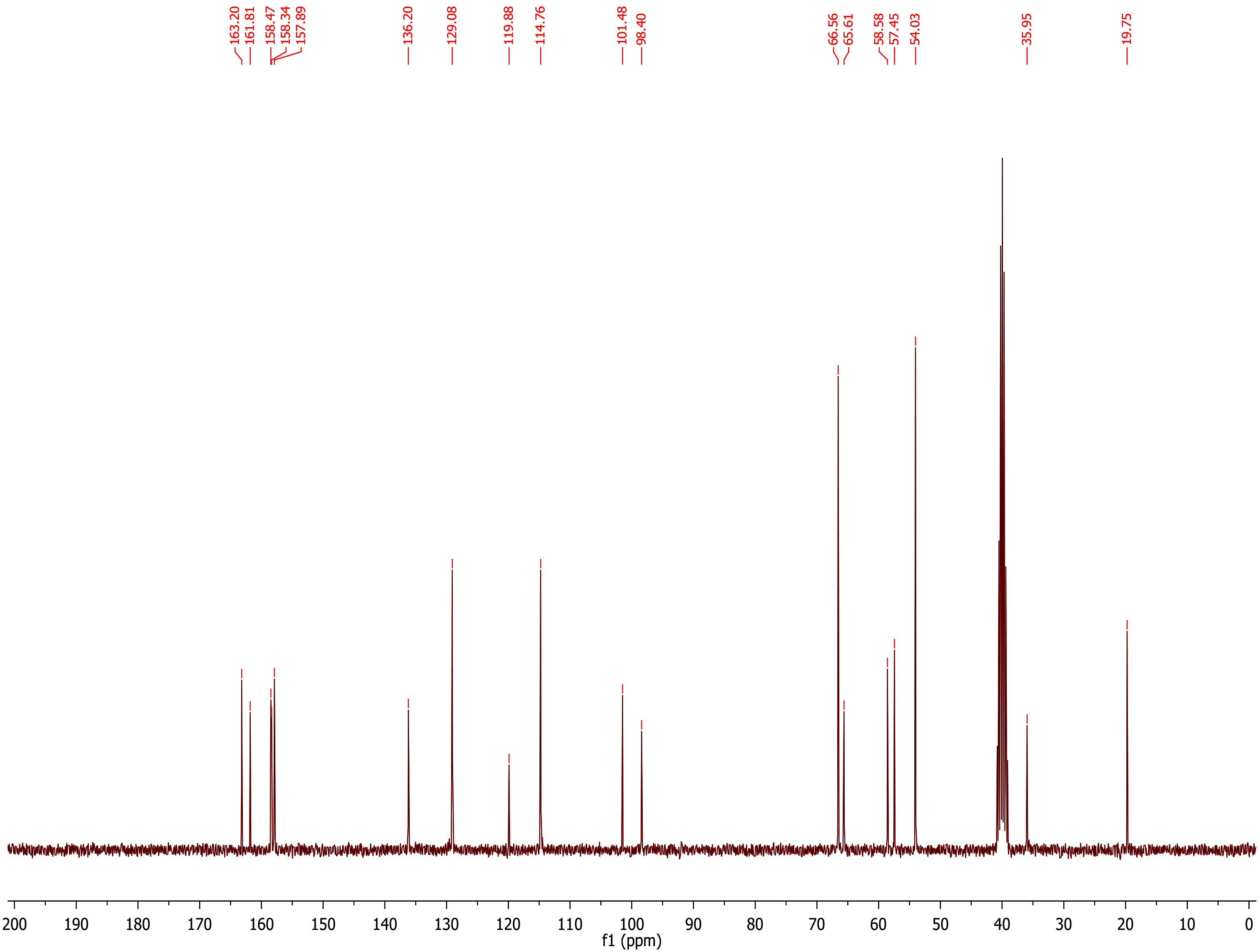


**Fig. S15.** ^13^C NMR spectrum of product **7f**

#
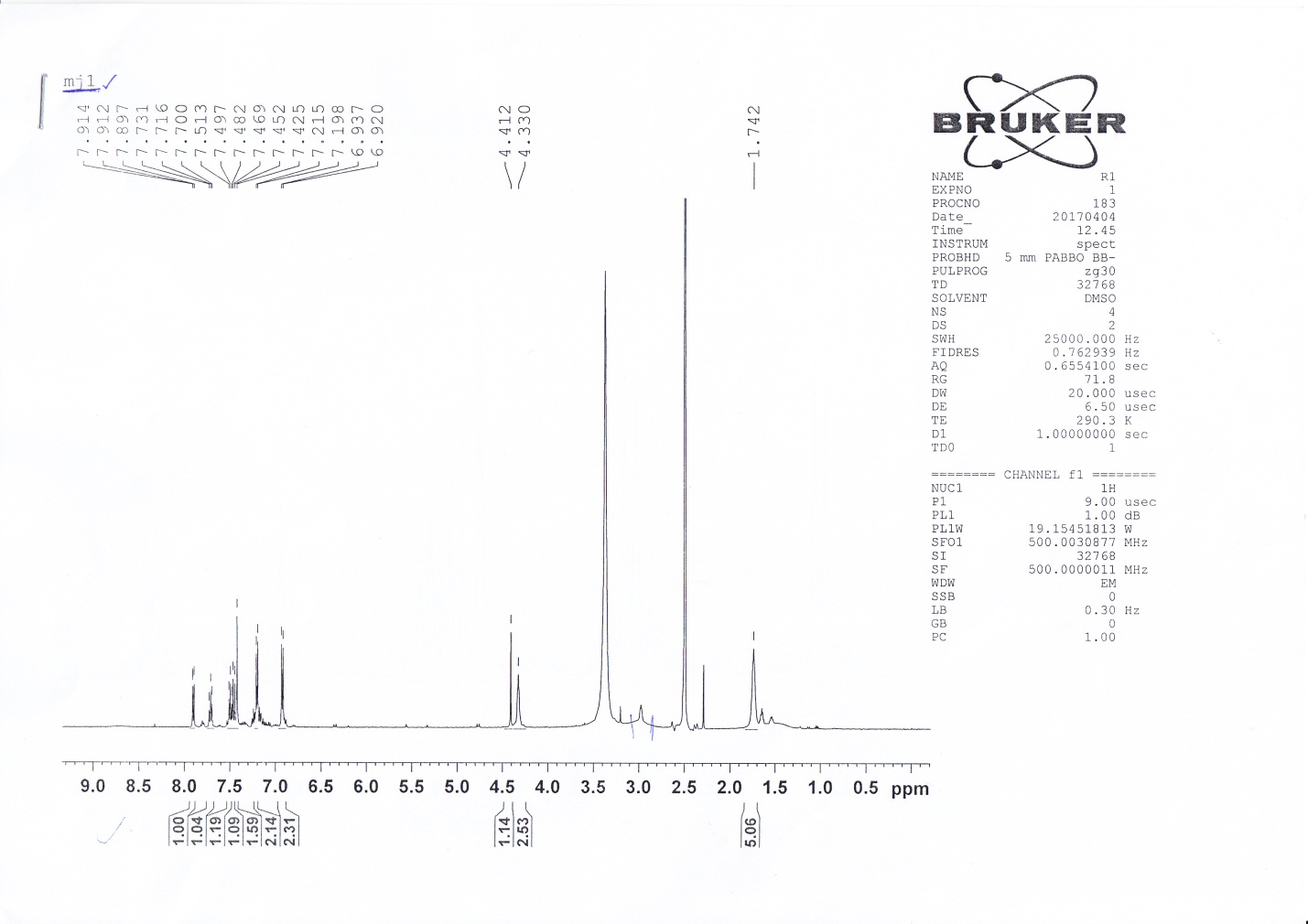


**Fig. S16.** ^1^H NMR spectrum of product **7g**

#
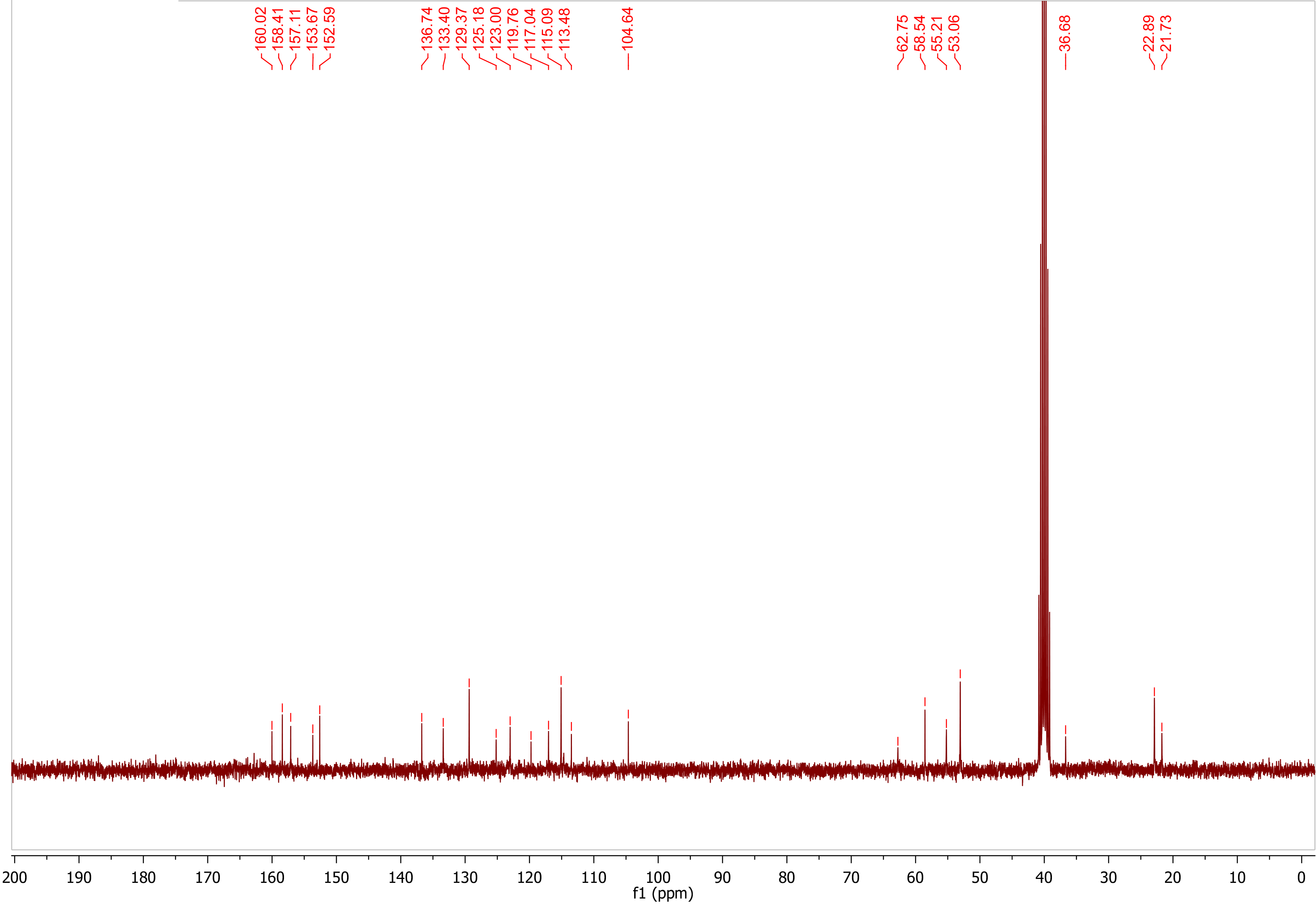


**Fig. S17.** ^13^C NMR spectrum of product **7g**

#
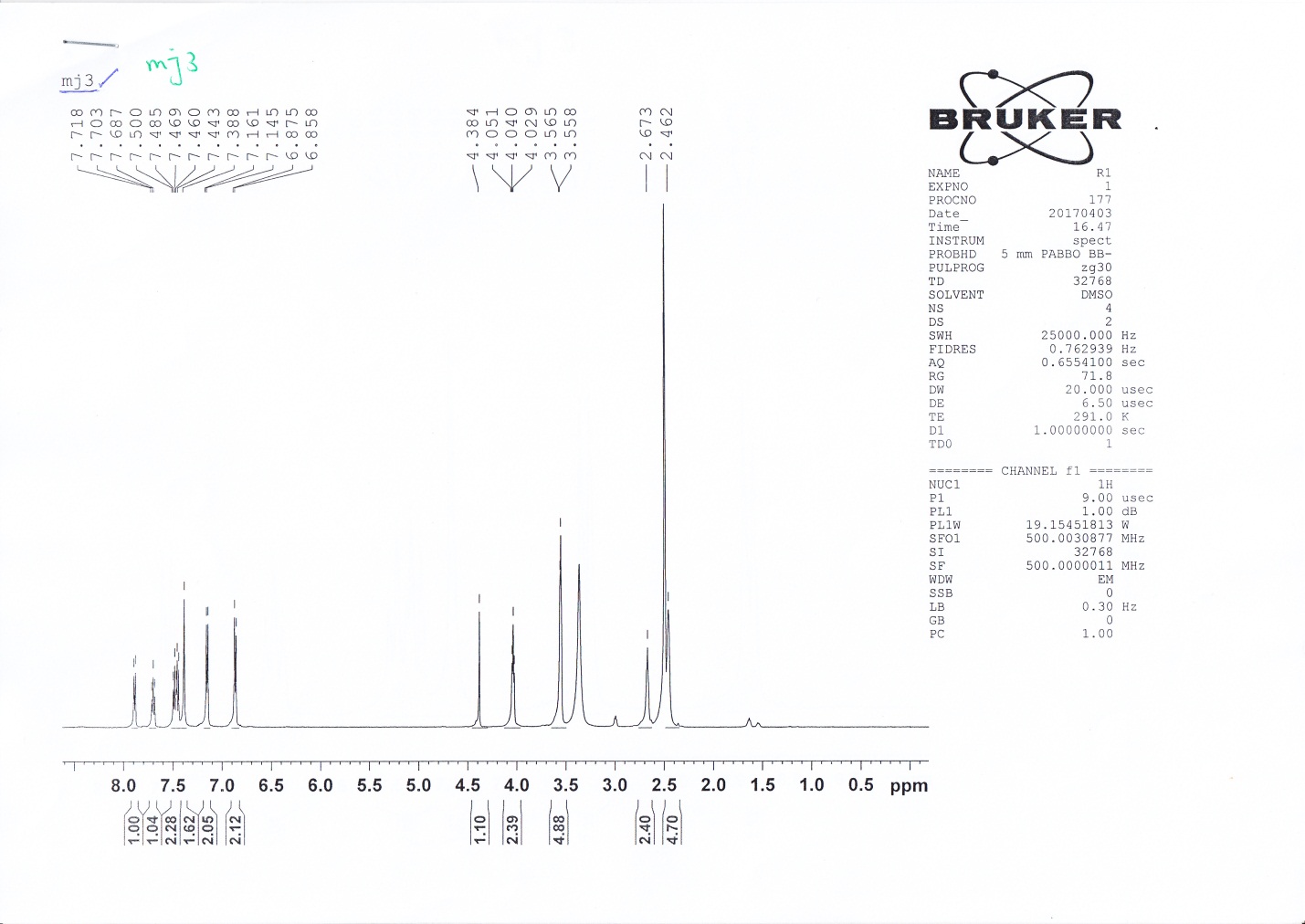


**Fig. S18.** ^1^H NMR spectrum of product **7h**

#
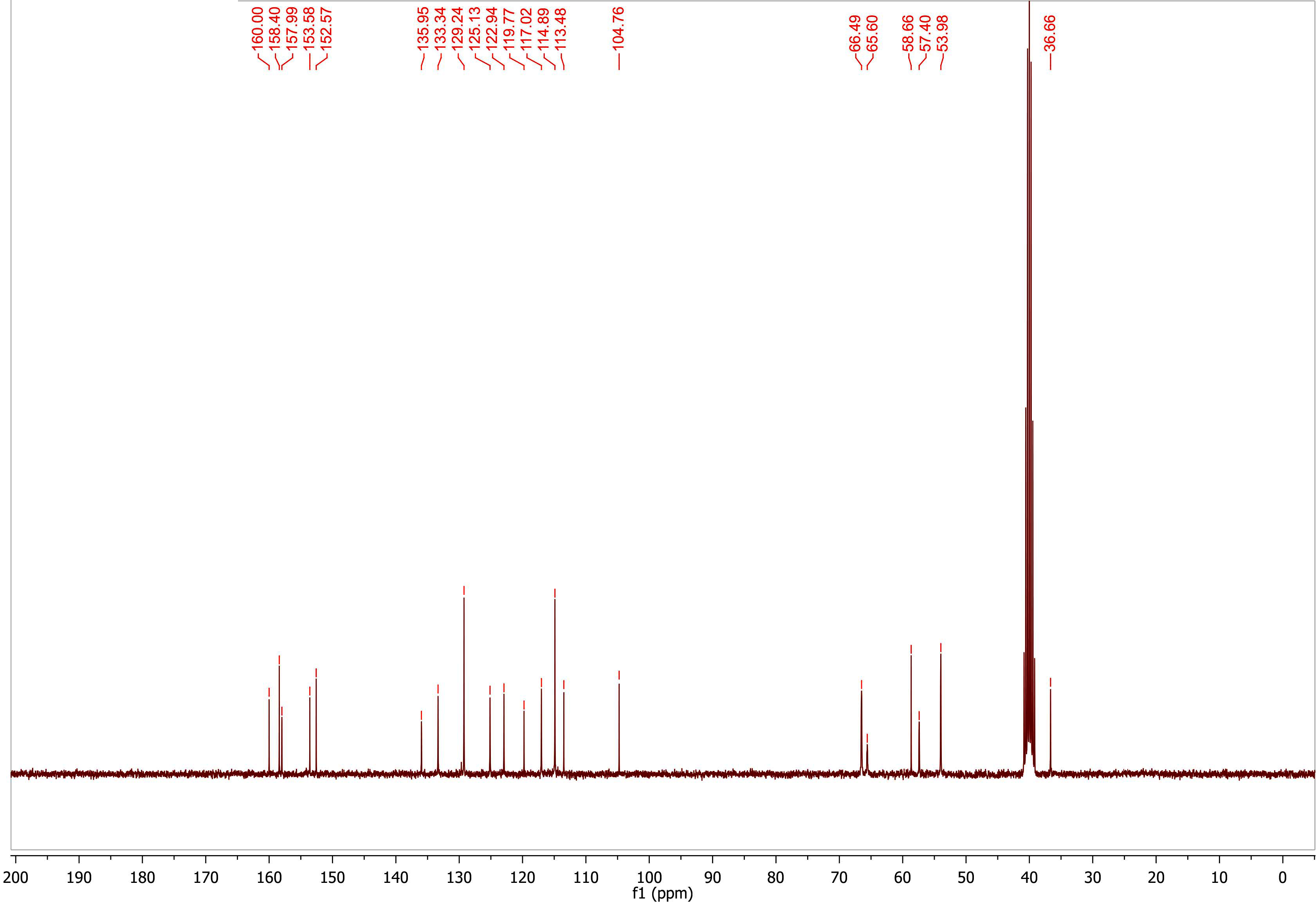


**Fig. S19.** ^13^C NMR spectrum of product **7h**
